# A Murine Model of Maternal Micronutrient Deficiencies and Gut Inflammatory Host-microbe Interactions in the Offspring

**DOI:** 10.1016/j.jcmgh.2024.01.018

**Published:** 2024-02-01

**Authors:** Ravi Holani, Paula T. Littlejohn, Karlie Edwards, Charisse Petersen, Kyung-Mee Moon, Richard G. Stacey, Tahereh Bozorgmehr, Zachary J. Gerbec, Antonio Serapio-Palacios, Zakhar Krekhno, Katherine Donald, Leonard J. Foster, Stuart E. Turvey, B. Brett Finlay

**Affiliations:** 1 Michael Smith Laboratories, University of British Columbia, Vancouver, British Columbia, Canada; 2 Department of Microbiology and Immunology, University of British Columbia, Vancouver, British Columbia, Canada; 3 Department of Medical Genetics, Faculty of Medicine, University of British Columbia, Vancouver, British Columbia, Canada; 4 British Columbia Children’s Hospital Research Institute, Vancouver, British Columbia, Canada; 5 Centre for Molecular Medicine and Therapeutics, University of British Columbia, Vancouver, British Columbia, Canada; 6 Department of Pediatrics, University of British Columbia, Vancouver, British Columbia, Canada; 7 British Columbia Children’s Hospital, Vancouver, British Columbia, Canada; 8 Biochemistry and Molecular Biology Department, University of British Columbia, Vancouver, British Columbia, Canada; 9 Department of Integrative Oncology, BC Cancer Research Institute, Vancouver, British Columbia, Canada

**Keywords:** *Enterobacteriaceae*, Maternal Micronutrient Deficiencies, Mitochondrial Dysfunction, Subclinical Inflammation

## Abstract

**Background & Aims:**

Micronutrient deficiency (MND) (ie, lack of vitamins and minerals) during pregnancy is a major public health concern. Historically, studies have considered micronutrients in isolation; however, MNDs rarely occur alone. The impact of co-occurring MNDs on public health, mainly in shaping mucosal colonization by pathobionts from the *Enterobacteriaceae* family, remains undetermined due to lack of relevant animal models.

**Methods:**

To establish a maternal murine model of multiple MND (MMND), we customized a diet deficient in vitamins (A, B12, and B9) and minerals (iron and zinc) that most commonly affect children and women of reproductive age. Thereafter, mucosal adherence by *Enterobacteriaceae*, the associated inflammatory markers, and proteomic profile of intestines were determined in the offspring of MMND mothers (hereafter, low micronutrient [LM] pups) via bacterial plating, flow cytometry, and mass spectrometry, respectively. For human validation, *Enterobacteriaceae* abundance, assessed via 16s sequencing of 3-month-old infant fecal samples (n = 100), was correlated with micronutrient metabolites using Spearman’s correlation in meconium of children from the CHILD birth cohort.

**Results:**

We developed an MMND model and reported an increase in colonic abundance of *Enterobacteriaceae* in LM pups at weaning. Findings from CHILD cohort confirmed a negative correlation between *Enterobacteriaceae* and micronutrient availability. Furthermore, pro-inflammatory cytokines and increased infiltration of lymphocyte antigen 6 complex high monocytes and M1-like macrophages were evident in the colons of LM pups. Mechanistically, mitochondrial dysfunction marked by reduced expression of nicotinamide adenine dinucleotide (NAD)H dehydrogenase and increased expression of *NAD phosphate oxidase* (*Nox*) *1* contributed to the *Enterobacteriaceae* bloom.

**Conclusion:**

This study establishes an early life MMND link to intestinal pathobiont colonization and mucosal inflammation via damaged mitochondria in the offspring.

## Graphical abstract

**Figure:**
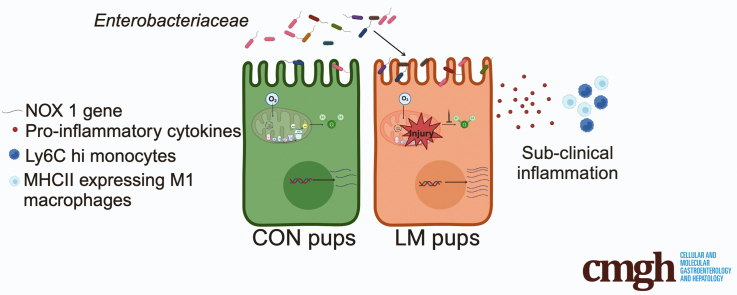

**Summary**

A novel mouse model of co-existing micronutrient deficiencies in pregnant mothers demonstrated the transgenerational consequences of micronutrient availability on colonization by pathobionts and sub-clinical inflammation in the gut.

An estimated 33% of the world population suffers from some form of malnutrition, defined as a dietary imbalance in the intake of nutrients including macronutrients (proteins, fats, and carbohydrates) or micronutrients (vitamins and minerals). Micronutrient deficiency (MND) rarely occurs alone and more frequently presents as multiple co-occurring deficiencies of vitamins and minerals.^1^^,^^2^ MNDs of the greatest public concerns, due to their significant impact on morbidity and mortality, are vitamin A, vitamin D, iron, zinc, folate (vitamin B9), vitamin B12, and iodine, notably among women of reproductive age and children under the age of 5 years.^1^ Although MNDs are more prevalent in low- and middle-income countries, industrialized nations are also susceptible as well due to the imbalanced diets, which are calorically high with low nutrient value.^2^, ^3^, ^4^, ^5^, ^6^, ^7^, ^8^ MND during pregnancy carries significant risks to the overall health of both the mother and the unborn fetus.^9^ Pregnant women are at increased risk of MNDs, as the estimated average requirement for most micronutrients increase substantially to support fetal growth and development.^10^, ^11^, ^12^ Furthermore, the developmental origins of health and disease theory suggests that perturbation in maternal nutrition such as MND during early life (first 2 years from conception) may skew gut microbial composition of the offspring towards an inflammatory phenotype.^13^ Whether maternal MNDs can affect intestinal colonization by pathobionts, such as *Enterobacteriaceae*, in the offspring remains to be explored.

*Enterobacteriaceae* is a family of bacteria encompassing potentially pathogenic commensals, also known as pathobionts.^14^ Enrichment of *Enterobacteriaceae* members is a known hallmark of dysbiotic intestines in malnourished children.^15^ Increased colonization by Enterotoxigenic *E. coli*, *Shigella,* and *Salmonella spp*. contributed to villous blunting, epithelial damage, and increased intestinal permeability in stunted Zambian children (<2 years old).^16^ Likewise, increased *Enterobacteriaceae* colonization in children from rural Zimbabwe resulted in environmental enteric dysfunction, a disease marked by intestinal inflammatory damage, loss of intestinal barrier, and microbial translocation to systemic sites.^17^ Furthermore, enhanced intestinal epithelial injury and turnover due to colonization by *Campylobacter jejuni* and Enteroaggregative *E. coli* may have contributed to reduced weight- and length-for-age ratios in children from Enteric Infections and Malnutrition and the Consequences for Child Health and Development (MAL-ED) cohort.^17^^,^^18^ Because most of the previous studies have focused on the contribution of either macronutrient malnourishment or MND in isolation, it remains unexplored whether early-life maternal MNDs (MMNDs) can shape the mucosal colonization of *Enterobacteriaceae* members and thus regulate intestinal inflammation in the offspring. Unfortunately, high-level investigation of MMNDs in humans is impossible for ethical reasons. Therefore, animal models can provide a biologically relevant alternative to investigate these intricacies.

This study describes a novel murine clinical model of MMND. We have further determined the impact of maternal micronutrients on the mucosal blooming of *Enterobacteriaceae* and on the intestinal inflammatory status of the offspring (F1) at weaning. In a validation study, we also confirmed a negative correlation between *Enterobacteriaceae* and micronutrient availability in human infants.

## Results

### Development of a Novel Maternal Murine MMND Model

**Figure 1:**
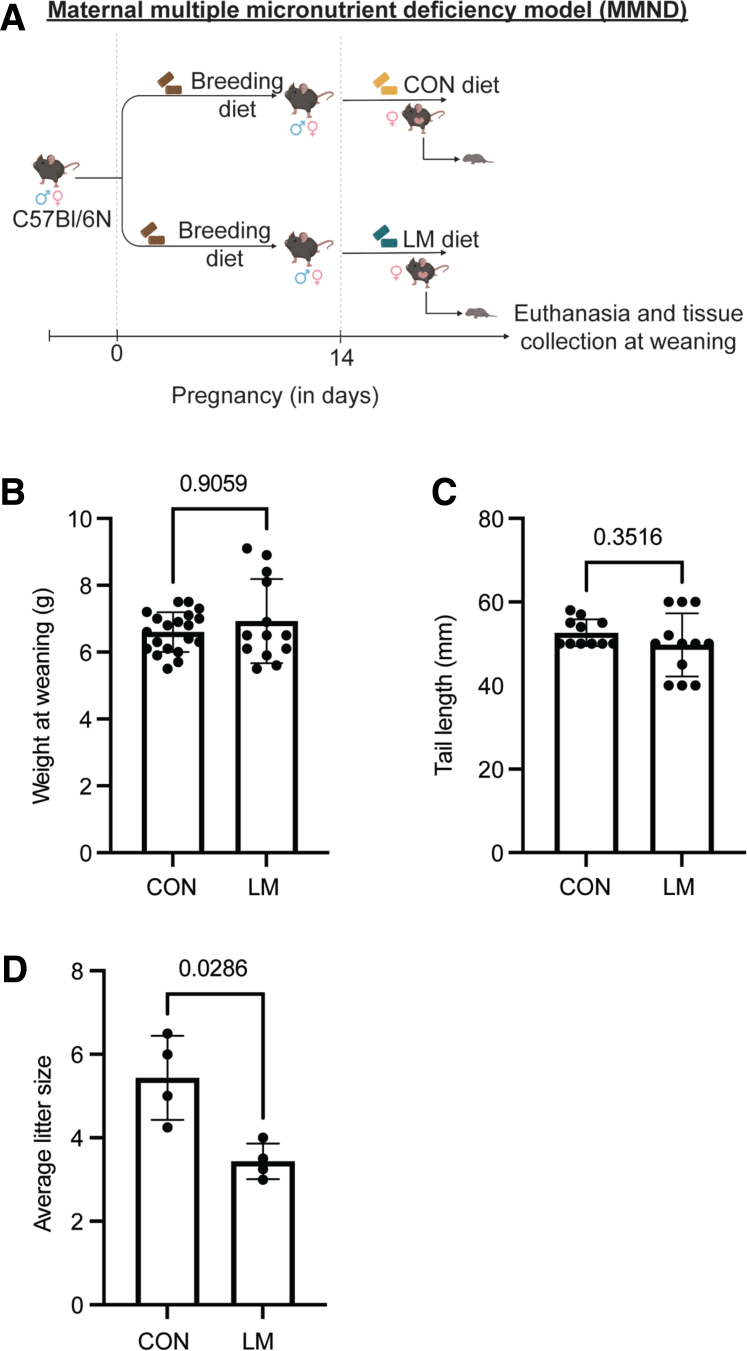
**Development and characterization of murine model of MMNDs.***A*, A schematic of the model. A bar graph representing weight in grams (*B*), tail length in mm (*C*), and average litter size (*D*) of pups at weaning in LM (∼31% males and 69% females) and CON (∼40% males and 60% females) groups. *D*, Litter size was ascertained by- ‘total pups/total mothers in respective CON or LM group’ for 4 different experiments. Data are mean ± standard deviation (n = 13–20 mice/group; 2 independent experiments) and *P* < .05 (Mann-Whitney test) was considered significant.

Co-occurring MMNDs during pregnancy are undeniably detrimental to child growth and development. Here, we developed a murine maternal MMND model using multiple micronutrients of public concern: vitamin A, vitamin B12, vitamin B9, zinc, and iron, via 2 different approaches (in Materials & Methods section and Figure 1*A*). For model 1, we noticed that feeding or administering the low micronutrient (LM) diets to female mice prior to mating led to either spontaneous abortion or cannibalization of pups soon after delivery (Table 1), underscoring the importance of micronutrient sufficiency in pregnant mothers. For model 2 (Figure 1*A*), we were able to successfully breed the mice on an LM diet, by first supplementing with a standard breeding diet, followed by the experimental diets at day 14 of pregnancy. The timeline of the newly developed model (ie, late gestation through weaning) was rationally adapted to remain appropriate for studying the host-microbe interactions at intestinal mucosa.^19^, ^20^, ^21^ Phenotypically, LM pups were characterized by reduced body hair with unnoticeable differences in weight (Figure 1*B*) and tail length (Figure 1*C*; as marker of stunting) compared with control (CON) pups. Although the role of malnourishment in reducing fertility rate or fecundity in mothers remains to be established,^22^ the specific role of maternal multiple MNDs on offspring health outcomes has not been explored either. In the newly developed MMND model, we determined lower fecundity in LM mothers with significantly smaller average litter size (∼3.5 pups) compared with CON mothers (∼5.5 pups) (Figure 1*D*). Overall, we showed a successful approach to developing a maternal murine MMND model to study host-microbe interactions.

**Table 1:** Pregnancy Outcome in MMND Model 1

Group	Bred	Aborted	Cannibalized
CON	6 of 6	0 of 6	0 of 6
LM	6 of 6	2 of 6	4 of 6

CON, Control; LM, low micronutrient; MMND, maternal murine model of multiple micronutrient deficiencies.

### MMND Promotes Enterobacteriaceae Abundance in Colons of LM Offspring

**Figure 2:**
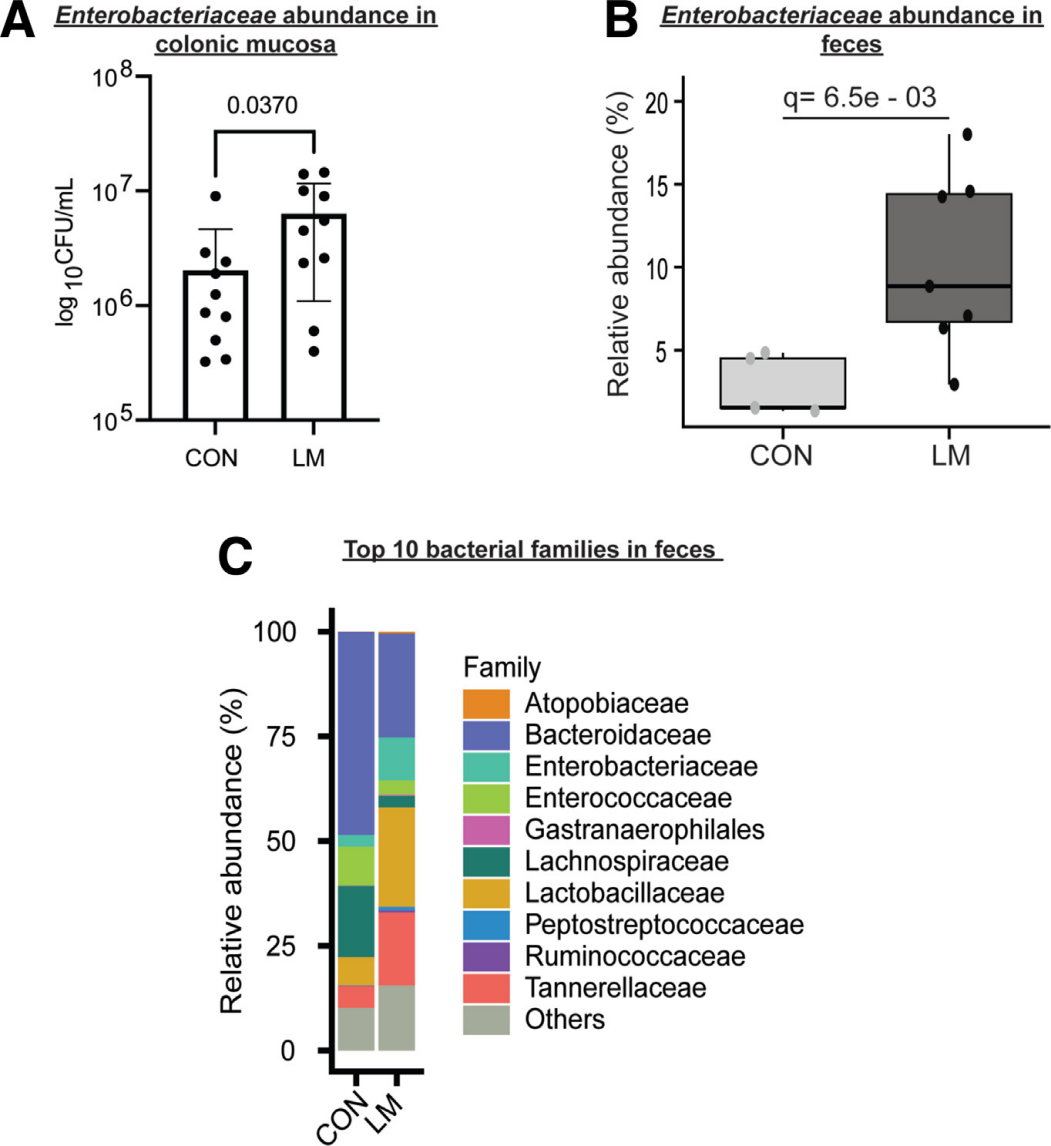
**MMNDs lead to mucosal colonization by *Enterobacteriaceae* in the colon of LM offspring.***A–B*, Bar plots representing *Enterobacteriaceae* (*A*) colony forming unit (CFU) count in colonic mucosa as log_10_ CFU/mL, normalized to total tissue weight (n = 10–17 mice/group; 2 independent experiments), and *B*, relative abundance as derived via 16S rRNA sequencing of fecal pellets (n = 5–7 mice/group, repeated once) in CON and LM pups. *C*, A stacked bar plot representing top 10 differentially abundant bacterial families in LM and CON pups (n = 5–7 mice/group). Data are mean ± standard deviation, and *P* (Mann-Whitney test) or q (Benjamini-Hochberg correction) < .05 was considered significant.

**Figure 3:**
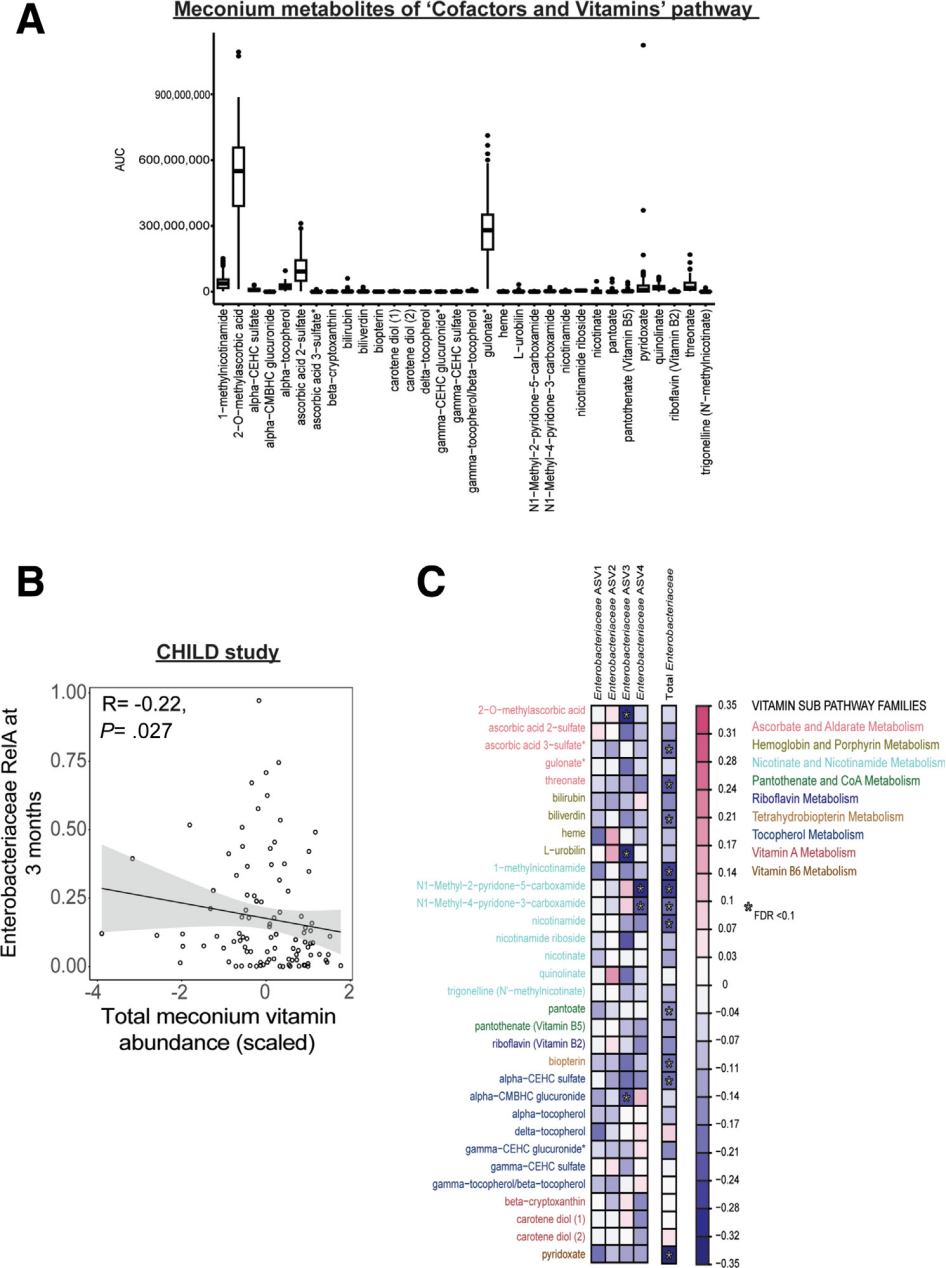
**Micronutrient-associated metabolites in meconium negatively correlate with the fecal *Enterobacteriaceae* abundance in CHILD cohort.***A*, A box plot representing AUCs for all the 32 metabolites from CHILD study. *B*, A scatter plot depicting a negative correlation between total vitamin levels in meconium of children at birth, and *Enterobacteriaceae* levels in feces at 3 months of age in CHILD cohort. *C*, A heat map representing correlations among vitamins/vitamin-associated metabolites and multiple amplicon sequencing variants (ASVs) of *Enterobacteriaceae*. A Spearman correlation test was performed, and *P* < .05 or q < 0.1 was considered significant (n = 100).

**Figure 4:**
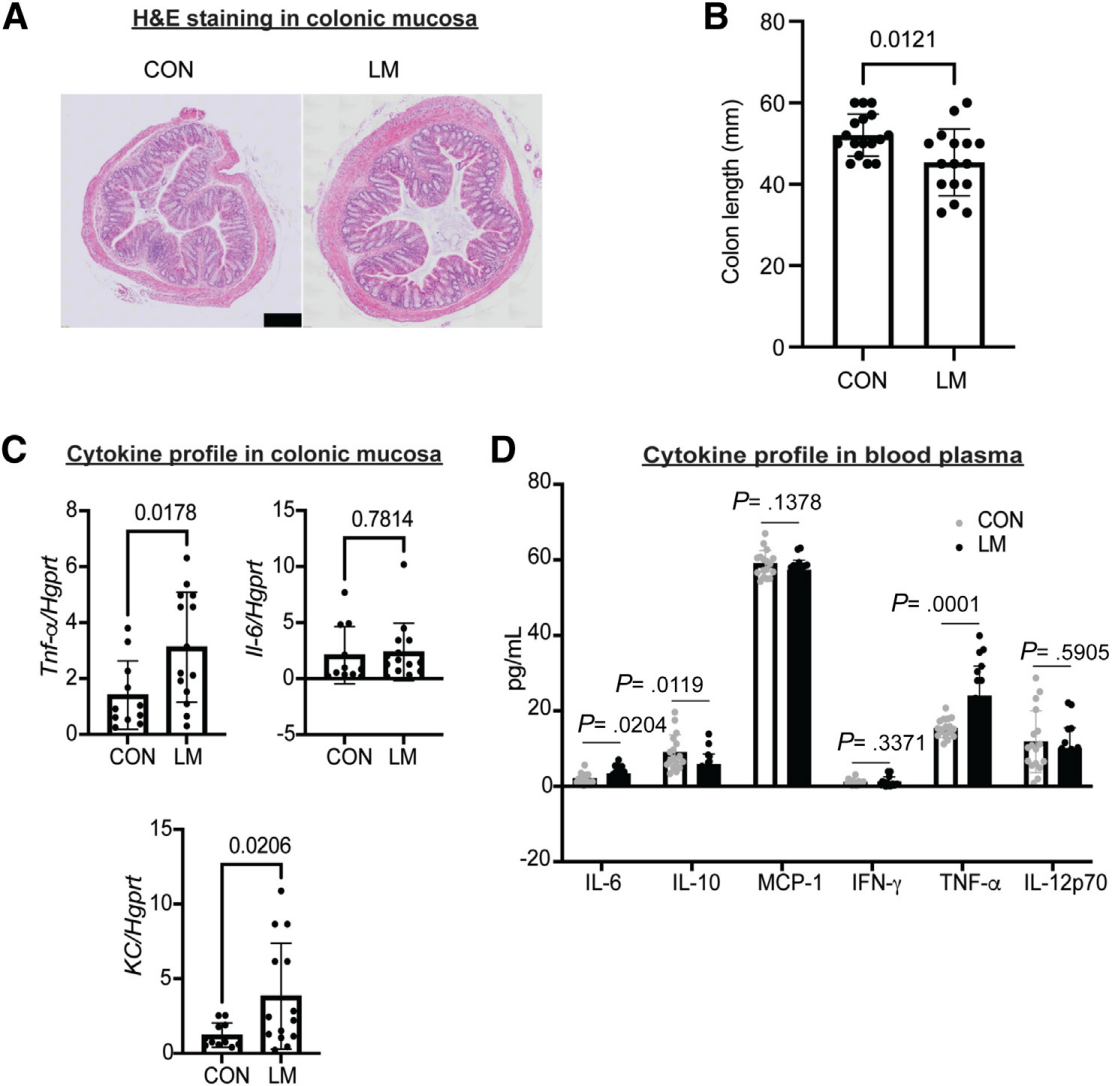
**MMNDs lead to subclinical inflammation in the colon of LM offspring.***A*, H&E microphotography of colons of CON and LM pups at 60X magnification (scale bar = 100 μm). *B*, A bar graph showing length of colons (in mm) from LM and CON pups at weaning. *C–D*, Expression of inflammatory cytokines in colons (*C*) and blood plasma (*D*) of CON and LM pups were determined by qPCR (*C*) or CBA bead-based assay (*D*). *C*, Data were normalized to *Hgprt* housekeeping gene. *D*, The data were quantitated by flow cytometry and analyzed using FlowJo software. Data are mean ± standard deviation (n = 10–17 mice/group; 2 independent experiments), and *P* < .05 (Mann-Whitney test) was considered significant.

**Figure 5:**
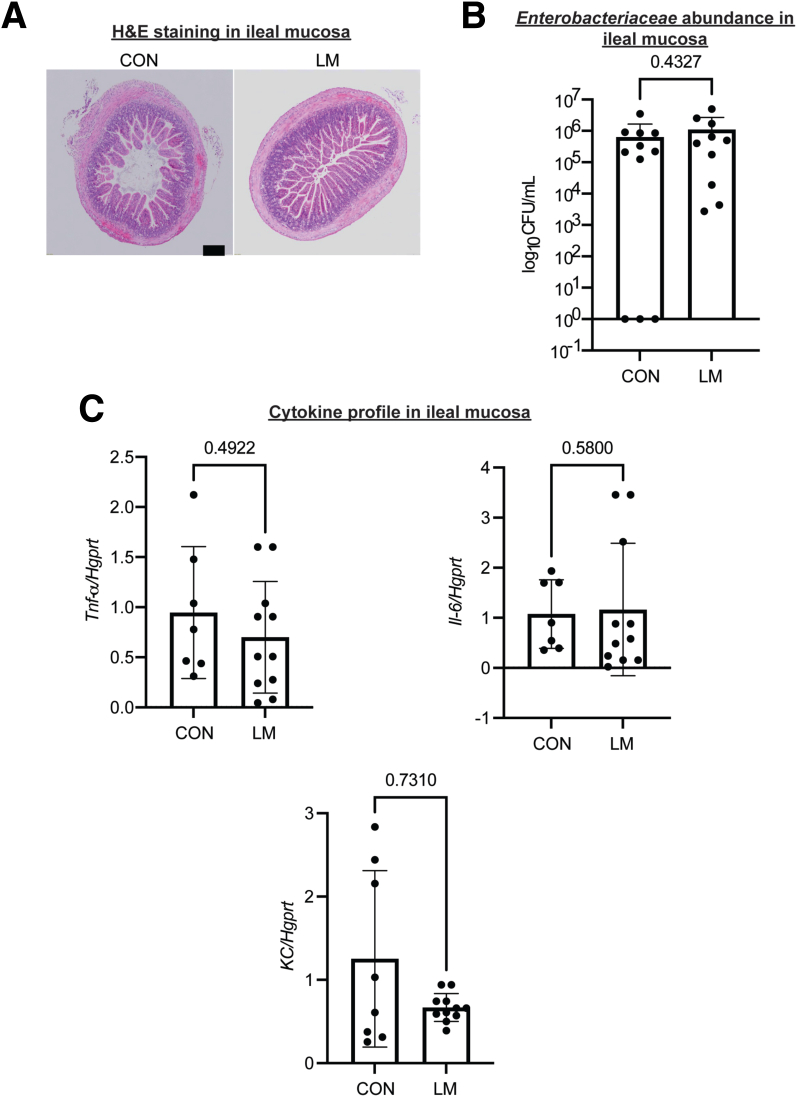
**Maternal MNDs do not promote *Enterobacteriaceae* colonization or inflammation in the ileum of the pups.***A*, H&E microphotography of ileums of CON and LM pups at 60× magnification (scale bar = 100 μm). *B*, A bar graph representing *Enterobacteriaceae* count in ileal mucosa of CON and LM pups as log_10_ CFU/mL, normalized to total tissue weight. *C*, Expression of pro-inflammatory cytokines (*Tnf-α, KC,* and *Il-6*) in ileums of CON and LM offspring as determined by qPCR. Data were normalized to *Hgprt* housekeeping gene. Data are mean ± standard deviation (n = 8–11 mice/group), and *P* < .05 (Mann-Whitney test) was considered significant.

Our lab has previously shown that malnourished diet deficient in proteins and fats increases barrier permeability and small intestinal abundance of *Enterobacteriaceae* in mice model of environmental enteropathy.^23^ However, the specific intergenerational role of MMND in promoting *Enterobacteriaceae* colonization in the offspring is understudied.^18^ In the newly developed MMND model, we determined an increase in the colonic mucosa-associated and fecal *Enterobacteriaceae* levels in the LM pups compared with CON pups at weaning (Figure 2*A* and 2*B*, respectively). Additionally, bacterial families including, *Atopobiaceae*, *Gastranaerophilales*, *Lactobacillaceae*, *Peptostreptococcaceae*, *Ruminococcaceae,* and *Tannerellaceae* were increased, whereas *Bacteroidaceae*, *Enterococcaceae*, and *Lachnospiraceae* decreased in the feces of LM vs CON pups (Figure 2*C* and Table 2). Next, using a subset of 100 children from the CHILD cohort, we sought to validate whether micronutrient availability within the gut has downstream impacts on *Enterobacteriaceae* blooming. We determined individual abundances of the 32 detected metabolites that belonged to co-factors and vitamins family (Table 3 and Figure 3*A*). Supporting our animal findings, we observed statistically significant negative correlations for total or some individual vitamin levels (r = −0.22; *P* = .027) in the meconium and *Enterobacteriaceae* abundance at 3 months of age (Figure 3*B* and 3*C*, respectively). Previous multi-cohort studies in children with moderate acute malnutrition have found elevated serum markers of inflammation, such as cytokines and C-reactive protein, with no overt signs of diseases.^24^^,^^25^ Similarly, we determined a sub-clinical inflammation in the intestine of LM pups characterized by no signs of gross pathology (Figure 4*A*); however, the colon length was relatively shorter than CON pups (Figure 4*B*). Furthermore, pro-inflammatory cytokines *Tnf-α* and *KC,* but not *Il-6,* expressions were enhanced in the colonic mucosa (Figure 4*C*). Next, we measured cytokine production in the blood plasma of LM and CON mice as a proxy for systemic inflammation. There was an increase in TNF-α and IL-6*,* a decrease in anti-inflammatory IL-10, and no change in monocyte chemoattractant protein-1, IFN-γ, and IL-12p70 cytokines in LM vs CON pups (Figure 4*D*). Interestingly, gross histopathology (Figure 5*A*), *Enterobacteriaceae* abundance (Figure 5*B*), and pro-inflammatory cytokine expressions (Figure 5*C*) were not statistically different in LM vs CON ilea. In conclusion, this novel MMND model primarily showed increased colonic abundance of *Enterobacteriaceae,* which was accompanied by a constitutive subclinical inflammation in the offspring.

**Table 2:** Differentially Abundant Bacterial Families in Feces of LM vs CON Pups

Family	Association	*P* value	q value
Lachnospiraceae	Negative	.003560818	0.008011841
Tannerellaceae	Positive	.023902671	0.041117848
Ruminococcaceae	Positive	4.95E-09	4.46E-08
Enterococcaceae	Negative	.025127574	0.041117848
Eggerthellaceae	Positive	1.38E-18	2.49E-17
Enterobacteriaceae	Positive	.002521347	0.006483465
Peptostreptococcaceae	Positive	4.38E-08	2.63E-07
Lactobacillaceae	Positive	7.48E-04	0.002243123
Rikenellaceae	Negative	.413549236	0.46524289
Erysipelotrichaceae	Negative	.363649339	0.436379207
Erysipelatoclostridiaceae	Negative	.21994483	0.28278621
Bacteroidaceae	Negative	.024269377	0.041117848
Akkermansiaceae	Positive	.044168527	0.065596702
Clostridiaceae	Negative	.047375396	0.065596702
Sutterellaceae	Negative	.832988526	0.832988526
Muribaculaceae	Positive	.78237105	0.828392877
Atopobiaceae	Positive	5.63E-05	2.03E-04
Gastranaerophilales	Positive	1.04E-05	4.70E-05

CON, Control; LM, low micronutrient.

**Table 3:** List of Metabolites From Cofactors and Vitamins Super Pathway

Biochemical	Sub Pathway
Pyridoxate	Vitamin B6 metabolism
Beta-cryptoxanthin	Vitamin A metabolism
Carotene diol (1)	Vitamin A metabolism
Carotene diol (2)	Vitamin A metabolism
Alpha-CEHC sulfate	Tocopherol metabolism
Alpha-CMBHC glucuronide	Tocopherol metabolism
Alpha-tocopherol	Tocopherol metabolism
Delta-tocopherol	Tocopherol metabolism
Gamma-CEHC glucuronide	Tocopherol metabolism
Gamma-CEHC sulfate	Tocopherol metabolism
Gamma-tocopherol/beta-tocopherol	Tocopherol metabolism
Biopterin	Tetrahydrobiopterin metabolism
Riboflavin (vitamin B2)	Riboflavin metabolism
Pantoate	Pantothenate and CoA metabolism
Pantothenate (vitamin B5)	Pantothenate and CoA metabolism
1-methylnicotinamide	Nicotinate and nicotinamide metabolism
N1-Methyl-2-pyridone-5-carboxamide	Nicotinate and nicotinamide metabolism
N1-Methyl-4-pyridone-3-carboxamide	Nicotinate and nicotinamide metabolism
Nicotinamide	Nicotinate and nicotinamide metabolism
Nicotinamide riboside	Nicotinate and nicotinamide metabolism
Quinolinate	Nicotinate and nicotinamide metabolism
Trigonelline (N'-methylnicotinate)	Nicotinate and nicotinamide metabolism
Bilirubin	Hemoglobin and porphyrin metabolism
Biliverdin	Hemoglobin and porphyrin metabolism
Heme	Hemoglobin and porphyrin metabolism
L-urobilin	Hemoglobin and porphyrin metabolism
2-O-methylascorbic acid	Ascorbate and aldarate metabolism
Ascorbic acid 2-sulfate	Ascorbate and aldarate metabolism
Ascorbic acid 3-sulfate	Ascorbate and aldarate metabolism
Gulonate	Ascorbate and aldarate metabolism
Threonate	Ascorbate and aldarate metabolism

Note: Super pathway for all metabolites = cofactors and vitamins.

### Ly6C ii Monocytes and M1-like Macrophages are Recruited in the Colon of LM Pups

**Figure 6:**
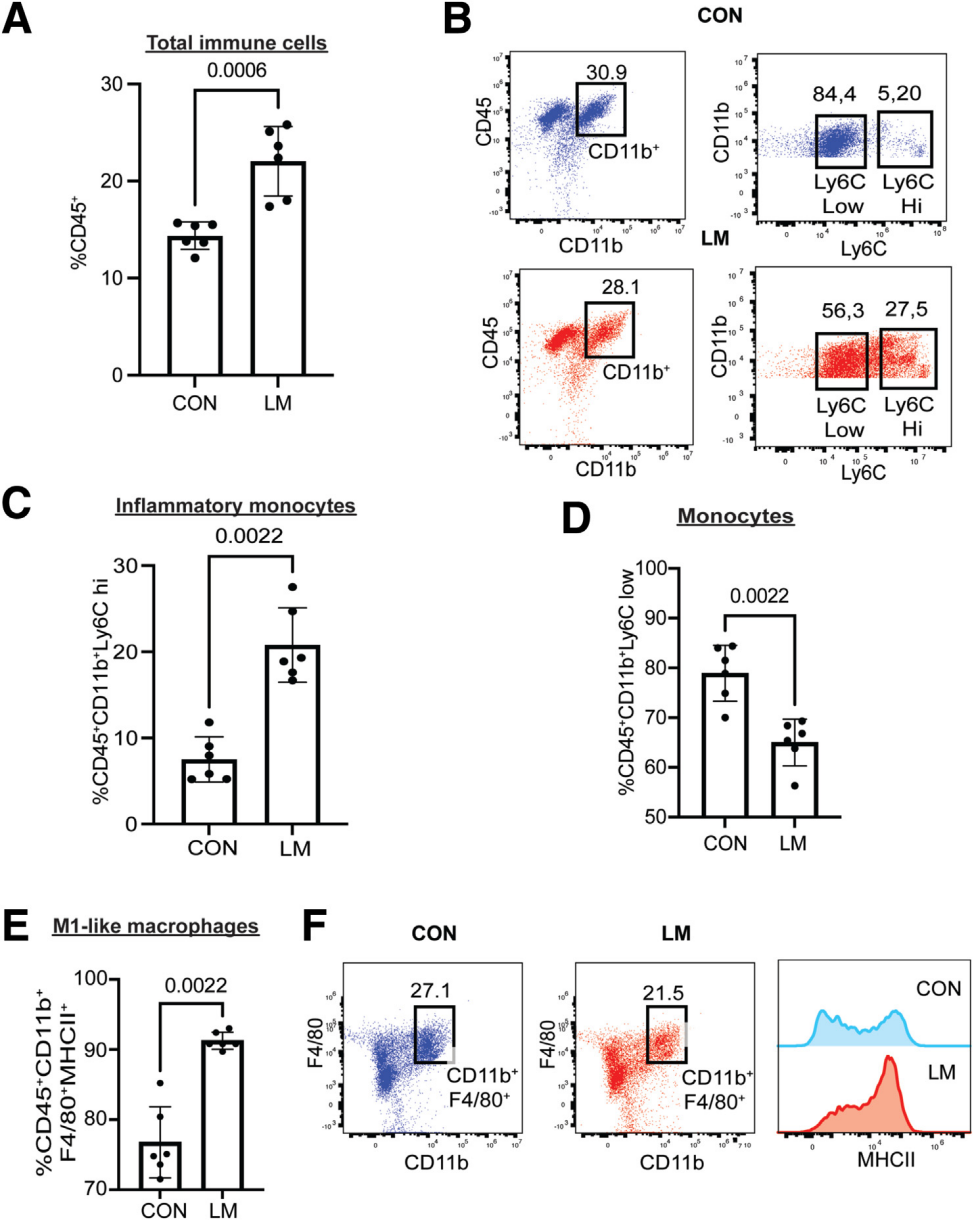
**MMNDs increase recruitment of total immune cells, Ly6C hi monocytes, and inflammatory M1 macrophages in the colons of the pups.***A–F*, Dot plots and graphs showing relative proportions of CD45^+^ (*A*), CD45^+^CD11b^+^Ly6C hi (*B* and *C*), CD45^+^CD11b^+^Ly6C low (*B* and *D*), and CD45^+^CD11b^+^F4/80^+^MHCII^+^ (*E* and *F*) cells in colonic mucosa of LM and CON pups. The data were quantitated by flow cytometry and analyzed using FlowJo software. Data are mean ± standard deviation (n = 6 mice/group), and *P* < .05 (Mann-Whitney test) was considered significant.

**Figure 7:**
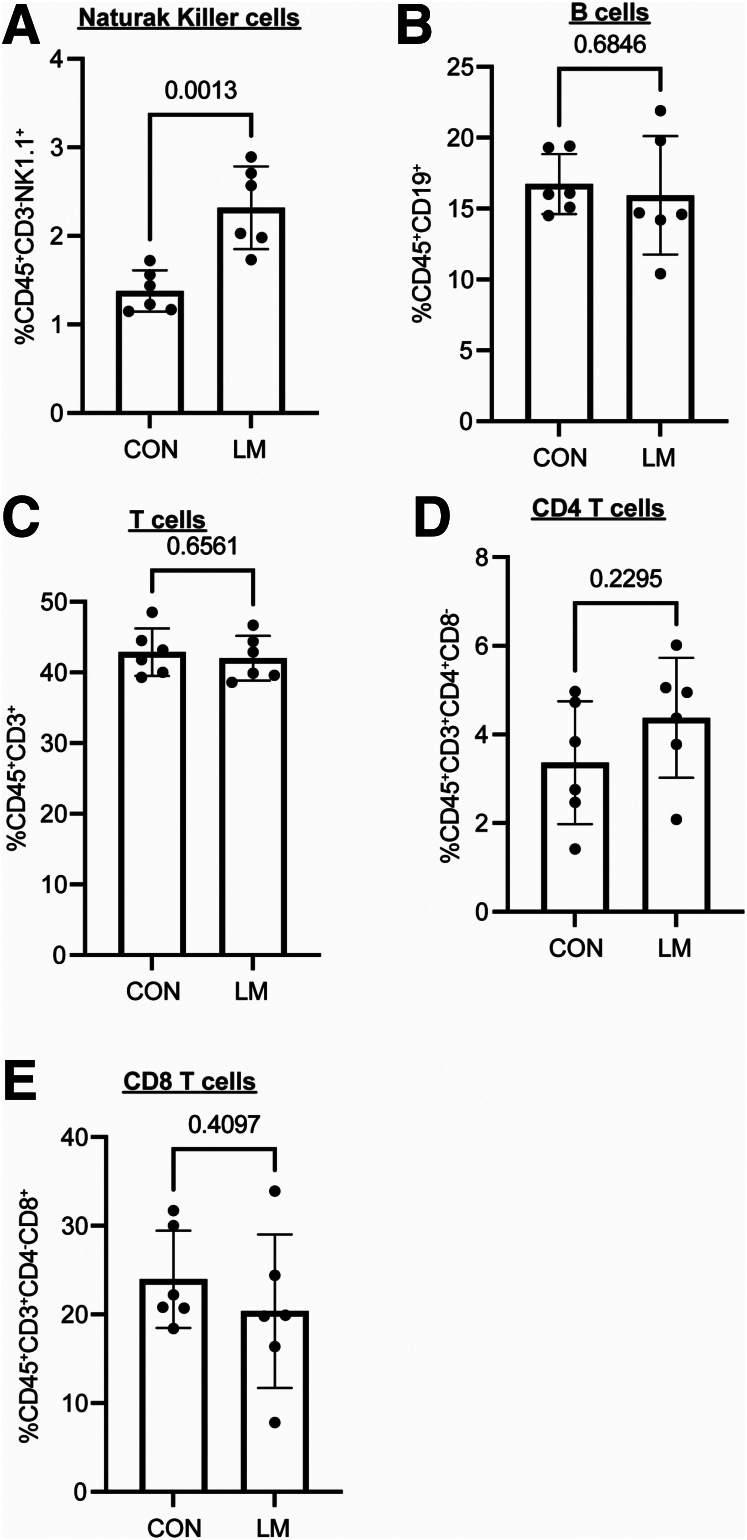
**MMNDs modulate recruitment of various immune cells in the colons of the LM pups.***A–H*, Graphs showing relative proportions of, CD45^+^CD3^-^NK1.1^+^ (*A*), CD45^+^CD19^+^ (*B*), CD45^+^CD3^+^ (*C*), CD45^+^CD3^+^CD4^+^CD8^-^ (*D*), and CD45^+^CD3^+^CD4^+^CD8^+^ (*E*) cells in colonic mucosa of LM and CON pups. The data were quantitated by flow cytometry and analyzed using FlowJo software. Data are mean ± standard deviation (n = 6 mice/group), and *P* < .05 (Mann-Whitney test) was considered significant.

Since we observed increased colonization by *Enterobacteriaceae,* specifically in LM colons, we next determined the immune cell profile at the colonic mucosa in the newly developed MMND model. We noticed an overall increase in colonic infiltration by immune cells (CD45^+^) (Figure 6*A*), suggesting a constitutive inflammatory stimulus in LM colon. Poor activation ability of helper (CD4^+^) and killer (CD8^+^) T-cells, reduced antibody productions, and increased pro-inflammatory monocyte/macrophage populations have routinely been reported in malnourished individuals.^26^, ^27^, ^28^ Of interest, MNDs may promote differentiation of monocytic cells into pro-inflammatory phenotypes such as high Lymphocyte antigen 6 complex (Ly6C hi)-expressing cells, which are commonly recruited to the target tissue during chronic inflammation by a chemokine monocyte chemoattractant protein-1.^29^, ^30^, ^31^ In the novel MMND model, we noticed an increase in colonic proportions of pro-inflammatory monocytes (CD45^+^CD11b^+^Ly6C hi) with a corresponding decrease in Ly6C low monocyte population (CD45^+^CD11b^+^Ly6C mild) in LM vs CON pups (Figure 6*B–D*). Furthermore, a concomitant increase in colonic mucosal proportion of macrophages (CD45^+^CD11b^+^F4/80^+^) expressing major histocompatibility factor II (pro-inflammatory M1-like macrophages) was evident (Figure 6*E–F*). Additionally, we found that proportions of natural killer cells (CD45^+^CD3-NK1.1^+^), which are important in mounting anti-bacterial defense against enteric gram-negative bacteria,^32^ were increased in colons of LM vs CON pups (Figure 7*A*). Interestingly, populations of adaptive immune cells, including antibody-producing B cells (CD45^+^CD19^+^) (Figure 7*B*), total T-cells (CD45^+^CD3^+^) (Figure 7*C*), CD4 T-cells (CD45^+^CD3^+^CD4^+^CD8^-^) (Figure 7*D*), and CD8 T-cells (CD45^+^CD3^+^CD4^-^CD8^+^) (Figure 7*E*) remained relatively unchanged in LM pups compared with CON pups. Together, LM pups had a chronic inflammatory phenotype characterized by an increase in mainly innate immune cell types, mostly Ly6C hi monocytes and M1-like macrophages in the colonic mucosa.

### Mitochondrial Dysfunction in the Colon Contributes to the Mucosal Blooming of Enterobacteriaceae in LM Pups

**Figure 8:**
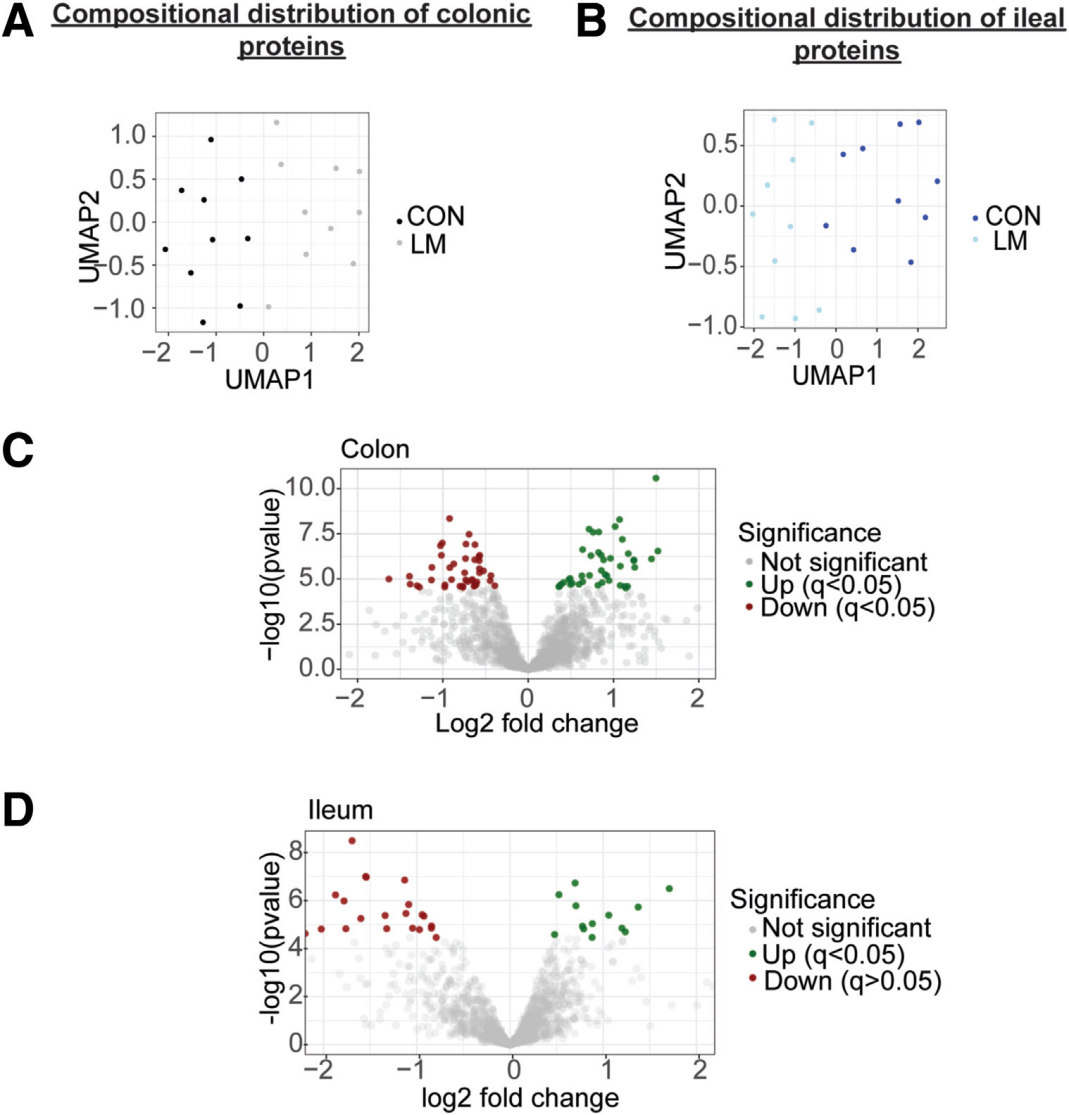
**MMNDs lead to altered global protein expressions in the intestines of the LM offspring.***A–B*, Uniform Manifold Approximation and Projection scatter plots showing spatial distribution of CON and LM groups based on proteomics profiling of colon (*A*) and ileum (*B*). *B–C*, Volcano plots depicting upregulated (*green*), downregulated (*red*), and unaltered (*gray*) proteins in colon (*B*) and ileum (*C*) of pups born to LM mother compared with corresponding CON controls. Data are mean ± standard deviation (n = 10 mice/group), and q value < 0.05 (limma package in R and Benjamini-Hochberg correction) was considered significant.

**Figure 9:**
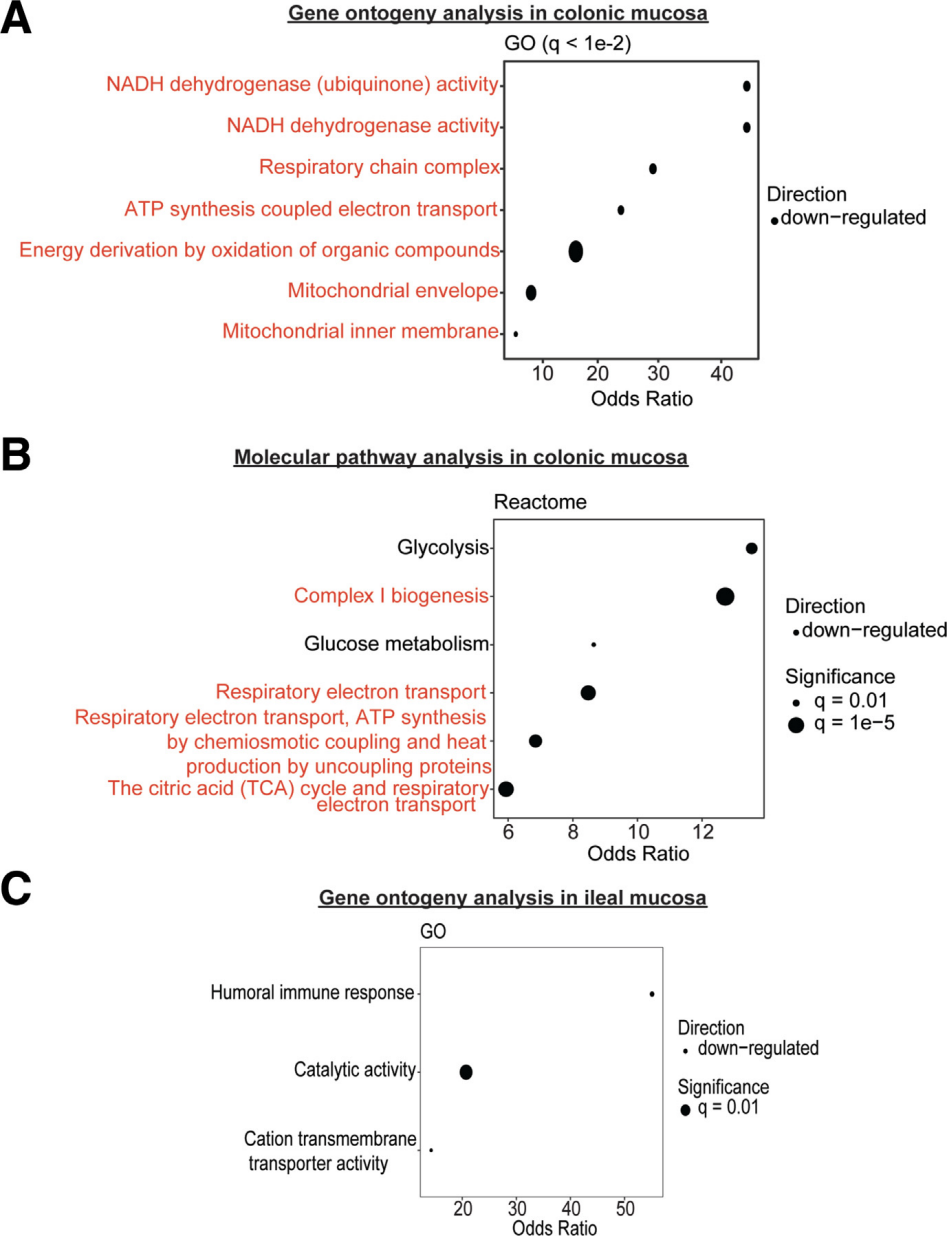
**MMNDs induce mitochondrial dysfunction in the colons but not ilea of the LM pups.***A–B*, GO (top 7 groups) (*A*) and Reactome (*B*) analyses showing gene groups (*A*) and molecular pathways (*B*) that were relatively downregulated in the colons of LM pups compared with the CON group. Mitochondria-related terms are highlighted in *red*. *C*, GO analysis showing differentially downregulated gene groups in the ilea of LM vs CON groups. Data are mean ± standard deviation (n = 10 mice/group), and q value < 0.05 (hypergeometric test and Benjamini-Hochberg correction) was considered significant.

**Figure 10:**
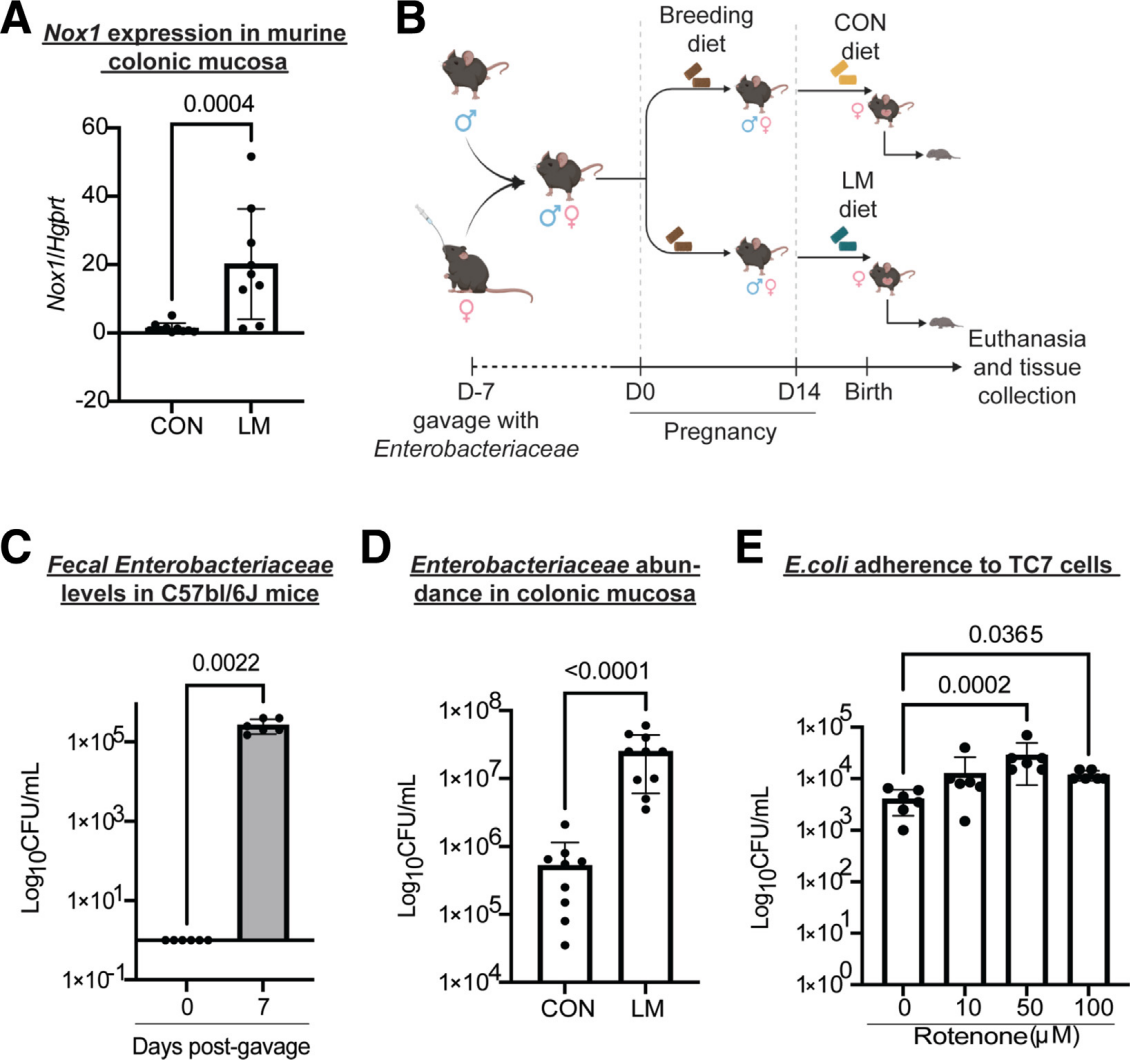
**Intestinal mitochondrial dysfunction contributes to *E. coli* attachment to the epithelium.***A*, *Nox1* gene expression was quantified in colons of C57Bl/6N LM and CON pups using qPCR. *Hgprt* was used as housekeeping control. *B*, A schematic of the MMND experiment with C57Bl/6J mice. *C–D*, Bar plots representing *Enterobacteriaceae* colony forming unit (CFU) count in colonic mucosa of C57Bl/6J female parents at Day 0 and 7 post-gavage (*C*) and C57Bl/6J CON and LM pups at weaning (*D*) as log_10_ CFU/mL, normalized to total tissue weight. *E*, Human colonic TC7 cells were pre-treated with rotenone at variable doses (as indicated) for 3 hours, followed by infection with commensal *E. coli* for 2 hours (n = 2, in triplicates). A bar graph representing absolute count of *E. coli* as log_10_ CFU/mL. Data are mean ± standard deviation (n = 9–10 mice/group, unless mentioned otherwise in respective figure), and *P* value < .05 (Mann-Whitney or Kruskal-Wallis test) was considered significant.

**Figure 11:**
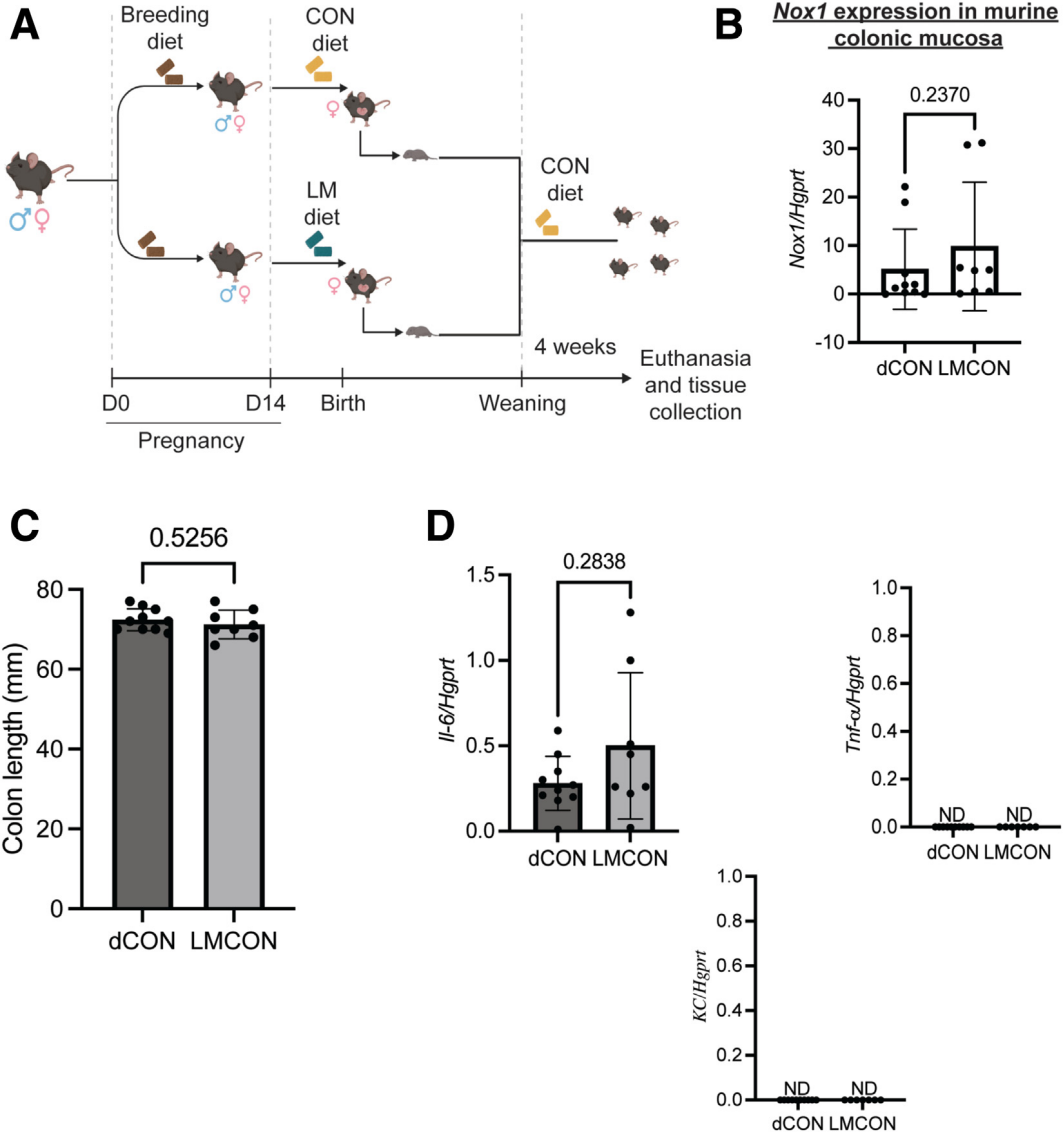
**Reversal of MMNDs lead to normalization of *Nox1* expression and inflammatory markers in the colons of LM offspring.***A*, A schematic of the diet reversal model in mice. *B*, *Nox1* gene expression was quantified in colons of dCON and LMCON pups using qPCR. *C*, A bar graph showing length of colons (in mm) from dCON and LMCON mice. *D*, Expression of pro-inflammatory cytokines *Il-6* , *Tnf-α,* and *KC* in colons of dCON and LMCON mice as determined by qPCR. *B* and *D*, *Hgprt* was used as housekeeping control. Data are mean ± standard deviation (n = 8α10 mice/group), and *P*-value < .05 (Mann-Whitney test) was considered significant.

**Figure 12:**
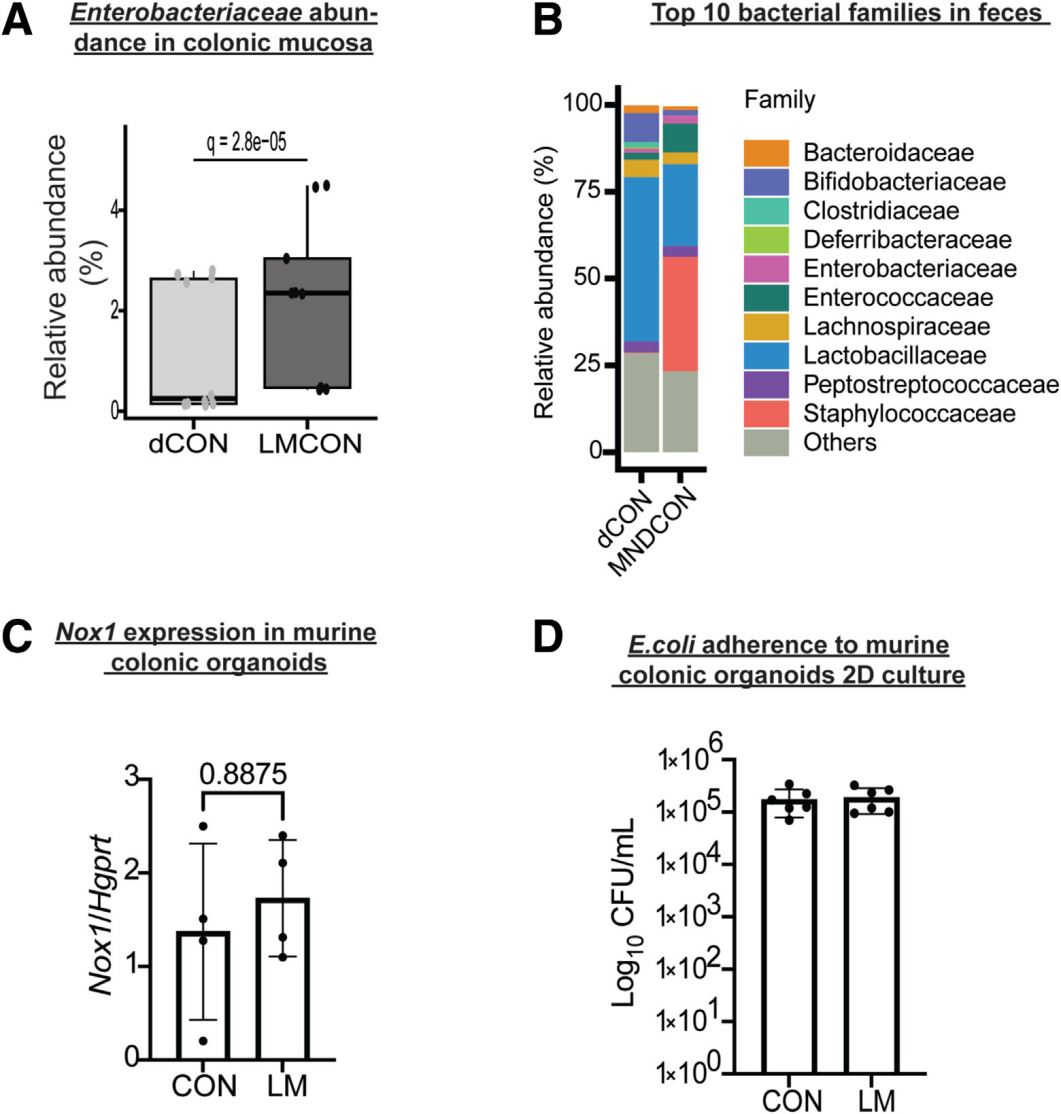
**Reversal of MMNDs did not normalize increased *Enterobacteriaceae* in the colon of LM offspring but reversed *E.coli* attachment in colonic organoids.***A*, A bar graph representing relative abundance of *Enterobacteriaceae* derived via 16S rRNA sequencing of fecal pellets from dCON and LMCON pups. *B*, A stacked bar plot representing top 10 differentially abundant bacterial families in dCON and LMCON pups. *C* and *D*, *Nox1* gene expression (*C*), and absolute count of *E. coli* as log_10_ CFU/mL (*D*) in colonic organoids from LM and CON pups (n = 4–6 mice/group). *Hgprt* was used as housekeeping control. Data are mean ± standard deviation (n = 8–10 mice/group, unless mentioned otherwise in respective figure), and *P*- or q-value < .05 (Mann-Whitney or Kruskal-Wallis test) was considered significant.

Next, we performed data-dependent acquisition proteomics to determine potential mechanisms of enhanced colonization by *Enterobacteriaceae* in the newly developed MMND model. We observed a distinct pattern of clustering for CON and LM groups on Uniform Manifold Approximation and Projection density plots for both colon and ileal proteins, confirming tissue-specific protein profiles (Figure 8*A–B*). Of 85 differentially abundant colonic proteins (q < 0.05), approximately one-half of them (42) were downregulated in the LM group (Figure 8*C* and Table 4). On the other hand, there were only 41 differentially abundant ileal proteins, of which 14 were upregulated and 27 were downregulated in LM vs CON groups (Figure 8*D* and Table 5). Gene ontogeny (GO) analysis identified that most protein clusters downregulated in LM colons belonged to mitochondrial functioning and bioenergetics, particularly related to nicotinamide adenine dinucleotide hydrogen (NADH) dehydrogenase/mitochondrial complex I and the electron transport chain (Figure 9*A*). Reactome-based molecular pathway analysis further confirmed dysfunctional mitochondrial and oxidative phosphorylation signaling in LM pups (Figure 9*B*). Although GO analysis in LM ilea only identified a total of 3 terms that passed FDR correction (Figure 9*C*), and there were no significant reactome-based molecular pathways, further suggesting that our novel MMND model has tissue-specific effects. Poor NADH dehydrogenase/mitochondrial complex I functioning can significantly enhance mitochondrial reactive oxygen species (ROS) production.^33^^,^^34^ Furthermore, mitochondrial ROS accumulation can activate nicotinamide adenine dinucleotide phosphate oxidase 1 (NOX1) enzyme, which is widely expressed in the plasma membrane of the intestinal epithelium and enhances oxygen-free radical synthesis in colonic cytoplasm and lumen.^35^ We confirmed that *Nox1* expression was upregulated in LM vs CON colonic mucosa (Figure 10*A*). Because increased ROS generation in gut lumen can selectively promote intestinal abundance of *Enterobacteriaceae* family,^36^^,^^37^ we tested whether mitochondrial dysfunction, and hence ROS generation, may promote attachment of commensal bacteria using two different models: (1) in vivo in C57BL/6J mice which are characterized by a spontaneous mutation in nicotinamide nucleotide transhydrogenase enzyme and abnormal ROS production^38^; and (2) in vitro in human colonic TC7 epithelial cells. We determined that C57Bl/6J mice were successfully colonized at day 7 post gavage with *Enterobacteriaceae* isolated from the feces of C57Bl/6N mice (Figure 10*B–C*). Next, we found that C57Bl/6J LM pups had strikingly higher levels of colonic mucosa-associated *Enterobacteriaceae* (∼2 log folds) (Figure 10*D*). Similarly, we found that inhibition of mitochondrial complex I by rotenone pre-treatment enhanced attachment of commensal *E. coli* to TC7 cells in a concentration-dependent manner (Figure 10*E*). Because micronutrient supplementation or fortification are commonly employed strategies to tackle MNDs in an affected population, we wanted to understand if a persistent nutrient-rich environment could reinstate mitochondrial functioning and prevent pathobiont colonization in LM pups. To do this, we developed a diet-reversal model, wherein the LM pups were transferred to the CON diet for 4 weeks after weaning (Figure 11*A*). We found that colonic *Nox 1* expression (Figure 11*B*) reverted to dCON level in LMCON pups. Similarly, colon length and pro-inflammatory cytokine *Il-6* expression in LMCON pups were statistically similar to dCON pups (Figure 11C–D, respectively), whereas expressions of *Tnf-a* and *KC* were not detected (Figure 11*D*). However, the relative abundance of *Enterobacteriaceae* was still higher in feces of LMCON vs dCON pups (Figure 12*A*), albeit to a lesser extent than LM C57Bl/6N pups at weaning (Figure 1*B*). Additionally, we noticed significant differences in abundance of other bacterial families, which are listed in Figure 12*B* and Table 6. Next, we isolated colonic crypts from LM pups and developed organoids ex vivo in a nutrient-rich media to simulate physiological environment. Though *Nox1* expression was consistent (Figure 12*C*), no quantitative difference in the *E. coli* attachment was evident in LM vs CON pups-derived organoid cultures (Figure 12*D*), which was contrary to the in vivo reversal experiment. Overall, poor maternal micronutrient availability damages mitochondria in the offspring by reducing NADH dehydrogenase expression. Consequently, luminal ROS synthesizing *Nox1* enzyme expression increases in the epithelium, which may enhance colonic abundance of *Enterobacteriaceae* in the offspring. Furthermore, persistent nutrient availability partially reverses the deleterious effects of MMND by reducing inflammation and minimizing *Enterobacteriaceae* colonization.

**Table 4:** A List of Differentially Abundant Colonic Proteins in LM Pups

Protein ID	Gene name	Effect size (log2)	*P*-value	q-value	Direction of change
Q9DC69	Ndufa9	−1.0171818698351500	4.9176643570658E-07	7.90268662180475E-04	Down-regulated
Q8BH04	Pck2	−0.5698744019772810	4.5456137025968E-06	0.007213888946021120	Down-regulated
P62889	Rpl30	0.38498391842565500	1.98783985519615E-05	0.031050058538163900	Up-regulated
Q9DC16	Ergic1	−0.7290496384786000	1.17047415233173E-07	1.89148623016808E-04	Down-regulated
Q91YT0	Ndufv1	−1.3020189692086000	2.33146903187949E-05	0.03627765813604490	Down-regulated
E9Q616	Ahnak	0.94758354213423000	1.21340442893264E-05	0.019086851667110500	Up-regulated
E9Q9C6	Fcgbp	1.2391346856646700	8.8119032098072E-07	0.0014090233232481700	Up-regulated
Q91VS7	Mgst1	0.5169999137432440	1.71667750067879E-05	0.026866002885623100	Up-regulated
O35074	Ptgis	−1.0277880624131500	1.43606521713443E-07	2.31780926045498E-04	Down-regulated
O35295	Purb	−0.7443545869244500	4.53387529502636E-06	0.00719979396850186	Down-regulated
O55126	Gbas	−0.6252080318095650	2.35180144316423E-05	0.03657051244120380	Down-regulated
O70400	Pdlim1	0.6393353942556060	1.4123260318744E-05	0.022159395440109300	Up-regulated
O70456	Sfn	0.901495129389748	5.64221767132574E-06	0.00894855722672263	Up-regulated
O88329	Myo1a	1.0781662909397500	2.26912480894703E-05	0.035330273275305200	Up-regulated
O88342	Wdr1	0.3587833745556320	2.68767102640811E-05	0.041578270778533400	Up-regulated
P00329	Adh1	1.144143516850340	3.17051783309298E-05	0.04892109016462470	Up-regulated
P00920	Ca2	0.9217420465877650	6.65085042176938E-06	0.010521645367239200	Up-regulated
P05202	Got2	−0.39110976834573700	2.35933415415862E-05	0.036664052755625000	Down-regulated
P06151	Ldha	0.7619493983424560	2.60699784756597E-08	4.22333651305687E-05	Up-regulated
P08207	S100a10	0.8172296887581710	2.22777437329802E-05	0.03470872473598320	Up-regulated
P09411	Pgk1	0.63829507239324	2.34658440979959E-07	3.78504065300674E-04	Up-regulated
P0DP28	Calml3	0.9650128495014000	7.18148488529362E-07	0.0011519101756011000	Up-regulated
P10639	Txn	1.0709637307592300	5.17442677382889E-09	8.40326908069812E-06	Up-regulated
P11688	Itga5	−0.7660890165059910	2.94283621103085E-05	0.04543739109831640	Down-regulated
P14824	Anxa6	−0.601833921491811	1.46513193834636E-05	0.022973268793271000	Down-regulated
P16110	Lgals3	0.8671999751166040	1.57784847876247E-05	0.02470910717742030	Up-regulated
P16460	Ass1	−1.273999972640000	2.8363554950416E-05	0.043821692398392800	Down-regulated
P16858	Gapdh;Gm3839	0.49179961132435600	9.34289493363338E-06	0.014761773995140700	Up-regulated
P17182	Eno1	0.4141117279066980	1.57279240854768E-05	0.024645657041942100	Up-regulated
P17751	Tpi1	0.4769660851008110	1.0744454679598E-05	0.016944005029726100	Up-regulated
P19324	Serpinh1	−0.8716923425011680	1.47080155918761E-06	0.0023473992884634200	Down-regulated
P22437	Ptgs1	−1.1283606740510700	2.28642441206065E-06	0.003642274088412610	Down-regulated
P24270	Cat	−1.3831652655920200	1.93923318169876E-05	0.030329606961768500	Down-regulated
P31786	Dbi	1.5202170499579300	2.85414602571919E-07	4.60088339345933E-04	Up-regulated
P42125	Eci1	−0.5755705444302770	5.83628353829163E-07	9.36723507895806E-04	Down-regulated
P47856	Gfpt1	1.019288940373460	1.24927660913274E-08	2.02757593662244E-05	Up-regulated
P48036	Anxa5	−0.6099245258318020	2.06676215445047E-05	0.032241489609427300	Down-regulated
P48758	Cbr1	0.6292103260257740	6.59438582685158E-06	0.010438912763906100	Up-regulated
P48962	Slc25a4	−0.6590987072353540	1.21812478751988E-05	0.019148921659812500	Down-regulated
P52196	Tst	−0.7320663029720220	1.12555269406664E-05	0.01772745493154960	Down-regulated
P52480	Pkm	0.7138800492018810	1.74193542393247E-08	2.82541925761846E-05	Up-regulated
P54071	Idh2	−0.5235004273740410	3.52845918071537E-06	0.005606721638156720	Down-regulated
P56391	Cox6b1	1.1037399123232500	6.44934726972134E-08	1.04350438824091E-04	Up-regulated
P84089	Erh	1.248695986080910	2.27717533297001E-06	0.0036298174807542000	Up-regulated
P99027	Rplp2	1.4979517453160000	2.63240828100999E-11	4.28292827320326E-08	Up-regulated
P99028	Uqcrh	1.4452349299947400	7.77795280650818E-07	0.0012460280396026100	Up-regulated
Q3UQ44	Iqgap2	1.237715263384300	9.73624453263659E-07	0.0015548782518620600	Up-regulated
Q91V92	Acly	1.1646027714965200	2.55782944481409E-05	0.039620778100170300	Up-regulated
Q68FD5	Cltc	0.8273326749240670	2.53546579080991E-08	4.10999004690286E-05	Up-regulated
Q62351	Tfrc	3.3549532892756100	4.49519458491449E-09	7.30918639507096E-06	Up-regulated
Q80X90	Flnb	0.8841179150567020	8.74911304281274E-07	0.0013998580868500400	Up-regulated
Q8BFR5	Tufm	−0.4368504158444770	6.4626739489949E-06	0.010236875535207900	Down-regulated
Q8BH59	Slc25a12	−0.6233638809428940	1.26535189211404E-07	2.04354330576417E-04	Down-regulated
Q8BMD8	Slc25a24	−0.574032620457047	9.61895563488708E-07	0.001537109110454960	Down-regulated
Q8BMK4	Ckap4	−0.9781753610744740	2.78969823242334E-05	0.043128734673264800	Down-regulated
Q8BWT1	Acaa2	−0.692756315751157	3.383847483537E-08	5.4784490758464E-05	Down-regulated
Q9WUA3	Pfkp	0.7189705613722490	6.29890048358158E-06	0.009983757266476800	Up-regulated
Q8K0C9	Gmds	0.7356656555620930	5.12874086820523E-07	8.23675783433759E-04	Up-regulated
Q8K3J1	Ndufs8	−1.0061373325053900	1.02419070768762E-07	1.65611637433088E-04	Down-regulated
Q8QZT1	Acat1	−0.571845237423549	2.66429838502315E-06	0.00423889873057183	Down-regulated
Q8R0Y6	Aldh1l1	1.173311316002950	3.97439816102578E-07	6.39878103925151E-04	Up-regulated
Q91V61	Sfxn3	−0.9213072890036610	4.51384641994155E-09	7.33500043240502E-06	Down-regulated
Q91VD9	Ndufs1	−1.3910446323582500	7.10073967628065E-06	0.011226269428199700	Down-regulated
Q91WD5	Ndufs2	−1.6323070402188000	1.00580034534228E-05	0.015881587452954600	Down-regulated
Q91WT7	Akr1c14	1.1302618764599300	2.57428488310453E-05	0.039849929990458100	Up-regulated
Q922Q1	Marc2	−0.7085594828559300	1.37694752110807E-05	0.021618076081396700	Down-regulated
Q99JY0	Hadhb	−0.6265326237259230	8.66169998318574E-07	0.0013867381673080400	Down-regulated
Q99KI0	Aco2	−0.6557037199393300	1.06588726954952E-05	0.016819701113491400	Down-regulated
Q99L13	Hibadh	−0.4454361110506540	1.24809544353259E-05	0.019607579417897000	Down-regulated
Q99LC3	Ndufa10	−0.9184319316054970	2.35818943668034E-06	0.0037542375831951100	Down-regulated
Q99N15	Hsd17b10	−0.5685713190996870	4.89526166226531E-07	7.87158075292262E-04	Down-regulated
Q9CPQ8	Atp5l	0.4954045300547160	2.02182453626773E-05	0.031560681011139300	Up-regulated
Q9CQA3	Sdhb	−0.9755228267565400	2.10735449918714E-05	0.032853656642327500	Down-regulated
Q9CQC7	Ndufb4	−0.7660451147218130	2.46096055910836E-05	0.03819410787736170	Down-regulated
Q9D0K2	Oxct1	−0.7341814642884000	7.40434837410179E-07	0.0011869170443685200	Down-regulated
Q9D154	Serpinb1a	0.369172207048607	2.43904252245649E-05	0.03787833037374930	Up-regulated
Q9D819	Ppa1	0.8554100243039610	4.79262454698919E-07	7.7113328961056E-04	Up-regulated
Q9DCS9	Ndufb10	−0.6338937050514080	2.47728623173612E-05	0.03842270945422720	Down-regulated
Q9DCT2	Ndufs3	−1.1335976585544300	1.14749848015516E-05	0.018061626077642300	Down-regulated
Q9EQ20	Aldh6a1	−0.7998924145386340	2.48728290588783E-05	0.03855288504126140	Down-regulated
Q9ERS2	Ndufa13	−0.8978429192454760	1.07889170036085E-05	0.017003333197686900	Down-regulated
Q9JKF1	Iqgap1	0.8249988991999700	3.40303292750374E-07	5.48228604620852E-04	Up-regulated
Q9QUH0	Glrx	0.5952811334929240	1.97719899559554E-05	0.030903620301158200	Up-regulated
Q9R100	Cdh17	0.8577598326879210	3.38306900834554E-06	0.005379079723269400	Up-regulated
Q9Z1Q9	Vars	1.080446902068260	1.9482174423313E-06	0.0031074068205184200	Up-regulated

LM, Low micronutrient.

**Table 5:** A List of Differentially Abundant Ileal Proteins in LM Pups

Protein ID	Gene name	Effect size (log2)	*P*-value	q-value	Direction of change
Q60605	Myl6	1.2370989878864900	2.01281730362787E-05	0.028883928307060000	Up-regulated
Q91YT0	Ndufv1	−0.8433293806317380	1.40842026326072E-05	0.020323504398852200	Down-regulated
E9Q509	Pklr	−1.1300153338196000	1.40375777454968E-07	2.05790889748983E-04	Down-regulated
Q6ZWZ6	Rps12	0.698585731327696	1.85930237916802E-07	2.72387798548114E-04	Up-regulated
F8VPQ6	Alpi	−4.004047567210490	1.83343230426363E-05	0.02632808788922580	Down-regulated
F8VPT3	Lct	−4.202408985704460	2.06092072667672E-06	0.0029986396573146300	Down-regulated
G3UZP7	H2-D1;H2-L	1.2005892358631700	1.40594705102753E-05	0.020301875416837600	Up-regulated
Q9R0P3	Esd	−1.7605285136884600	1.49006931828689E-05	0.021456998183331200	Down-regulated
O55142	Rpl35a	0.7074803931822540	1.65422220916579E-06	0.0024102017587545600	Up-regulated
O70475	Ugdh	−2.751201326462120	3.4236213696455E-09	5.03272341337889E-06	Down-regulated
O88312	Agr2	0.790212914668354	1.51591376337715E-05	0.02181399905499720	Up-regulated
P03930	Mtatp8	1.0597763357486900	4.05621400904059E-06	0.005889622741126940	Up-regulated
P12787	Cox5a	0.8836041756435050	9.14052870878679E-06	0.013235485570323300	Up-regulated
P24270	Cat	−1.693930533094730	3.22382719790435E-09	4.7422498081173E-06	Down-regulated
P24549	Aldh1a1	−1.0447106634966300	1.41518172923822E-05	0.020406920535615200	Down-regulated
P28271	Aco1	−0.7917686970933590	3.45645356990867E-05	0.04946185058539300	Down-regulated
P28843	Dpp4	−0.9711436627995890	1.62290410551764E-05	0.023321131996288500	Down-regulated
P29758	Oat	−0.9232173792610570	4.47535539220895E-06	0.0064892653187029700	Down-regulated
P34884	Mif	0.8806908186675840	3.43356753663742E-05	0.0491686871246479	Up-regulated
P34914	Ephx2	−2.93616928671433	2.83950119037278E-07	4.15702974270575E-04	Down-regulated
P48758	Cbr1	−1.3223961841851400	1.48001184186236E-05	0.02132697064123650	Down-regulated
P49290	Epx	−2.646435499246080	5.74808605994743E-07	8.39795373358319E-04	Down-regulated
P51660	Hsd17b4	−0.8419867889953800	1.1426957744384E-05	0.016523380898379300	Down-regulated
P52825	Cpt2	−1.7806838624063500	1.03831899808693E-06	0.00151490741820883	Down-regulated
P54869	Hmgcs2	−2.6987032108217300	1.09994149071065E-05	0.01591615337058310	Down-regulated
Q921G7	Etfdh	−1.0869424581697400	1.45685514603955E-06	0.0021240948029256600	Down-regulated
Q7M758	Naaladl1	−1.8716158073509200	5.8252018817094E-07	8.50479474729572E-04	Down-regulated
Q8BW75	Maob	−1.5478150075831100	1.00016052348323E-07	1.46823564847338E-04	Down-regulated
W4VSN6	Defa22;Defa21	2.425464679176370	1.2267908162544E-08	1.80215570907771E-05	Up-regulated
Q8K157	Galm	−1.59980361971755	5.58192983560826E-06	0.008088216331796370	Down-regulated
Q8VC30	Dak	−1.5423677136468100	1.06035568786154E-07	1.55554179409288E-04	Down-regulated
Q921I1	Tf	1.7093276560266400	3.1771181971545E-07	4.64812392243703E-04	Up-regulated
Q9CPQ8	Atp5l	0.7772787473575950	1.16516349550101E-05	0.01683661250998960	Up-regulated
Q9CQ19	Myl9	1.3754825179014200	1.86302361070536E-06	0.002712562377187010	Up-regulated
Q9CZX8	Rps19	0.5251096537064370	5.69304993283564E-07	8.32323900180571E-04	Up-regulated
Q9DBM2	Ehhadh	−0.942097479342048	3.87778797764859E-06	0.005634425931523400	Down-regulated
Q9EST5	Anp32b	0.47853237702428100	2.58439135165197E-05	0.03703432806917270	Up-regulated
Q9JIL4	Pdzk1	−2.02425223337327	1.53678379104192E-05	0.022098950915182800	Down-regulated

LM, low micronutrient.

**Table 6:** Differentially Abundant Bacterial Families in Feces of LMCON vs dCON Pups

Family	Association	*P* value	q_value
Rikenellaceae	Negative	.293886374	0.391848499
Streptococcaceae	Negative	.087369472	0.145615786
Tannerellaceae	Negative	.533401548	0.561475314
Enterobacteriaceae	Negative	4.18E-06	2.79E-05
Peptostreptococcaceae	Negative	.01571997	0.034933266
Marinifilaceae	Positive	.567382059	0.567382059
Lactobacillaceae	Positive	.021072556	0.042145113
Staphylococcaceae	Negative	1.29E-19	2.57E-18
Deferribacteraceae	Positive	1.41E-04	5.64E-04
Enterococcaceae	Negative	1.65E-08	1.65E-07
Erysipelatoclostridiaceae	Positive	.476459053	0.561475314
Bacteroidaceae	Positive	.005359202	0.013398006
Akkermansiaceae	Positive	.2113315	0.325125385
Bifidobacteriaceae	Positive	9.59E-04	0.00319821
Clostridiaceae	Positive	3.29E-05	1.64E-04
Sutterellaceae	Positive	.513729959	0.561475314
Erysipelotrichaceae	Positive	.520469479	0.561475314
Lachnospiraceae	Positive	.003475805	0.009930871
Eggerthellaceae	Positive	.032376401	0.058866183
Muribaculaceae	Negative	.25724017	0.367485957

dCON, CON pups continued on CON diet; LMCON, LM pups transferred to CON diet.

## Discussion

In this study, we developed a model of early life MMNDs, suitable for studying host-microbe interactions. Although we chose to focus on offspring microbiome and gut inflammation, this model is well-poised to aid the scientific community in future studies concerning the role of early life MND in adult metabolic diseases. We showed that inadequate maternal micronutrient availability increased the intestinal *Enterobacteriaceae* colonization and the associated gut and systemic inflammatory signature in the LM offspring. The inflammation was characterized by increased cytokine production and colonic infiltration of chronic Ly6C hi monocytes and pro-inflammatory M1-like macrophages. Lastly, mitochondrial dysfunction, particularly decreased in NADH dehydrogenase/mitochondrial complex I production, and increased ROS-producing *Nox1* enzyme expression coincided with *Enterobacteriaceae* blooming in the offspring colons.

Our model was based on a previous study from our lab, which characterized the role of micronutrients in host growth, metabolism, and gut microbiome in mice.^39^ Micronutrients interact to influence absorption and bioavailability of each other, and hence, co-existing and increased deficiencies in minerals and vitamins are likely.^1^^,^^39^, ^40^, ^41^, ^42^, ^43^ However, previous maternal animal models involving micronutrients are based on deficiency/supplementation of only 1 or 2 micronutrient(s) and are unable to interrogate the deleterious outcomes of multiple co-existing MNDs. A recent maternal model of single MND shows that gestational vitamin C deficiency induces aberrant DNA methylation in fetal germ-cell development, resulting in lower oocyte numbers and reduced fecundity rate in the female offspring.^44^ Likewise, a model of gastric intrinsic factor (Gif)-deficient C57Bl/6 mice, which lack the capacity to absorb vitamin B12, has previously been developed to study gut immunological and developmental changes in the offspring.^45^ Our MMND model not only combines multiple micronutrient deficiencies, but also captures the intergenerational effects of maternal malnutrition on pathobiont colonization and enteric inflammation. Furthermore, the LM pups did not exhibit any overt signs of stunting, such as reduced tail length and body weight, perhaps due to the specific time frame of the model and the absence of more severe forms of malnutrition.^39^^,^^46^

MMND substantially contributed to the intestinal blooming of *Enterobacteriaceae* and subclinical inflammation in the offspring. Pathobionts and colitogenic bacteria from the *Enterobacteriaceae* family, such as *Salmonella* and *Escherichia spp.*, are often over-represented in the intestines of children from malnourished settings.^1^^,^^37^ It has been inferred from the MAL-ED cohort that the prevalence of pathobionts in non-diarrheal stool of children from undernourished countries increases by more than 2-fold from birth to 8 to 24 months of age.^18^ Likewise, a recent re-analysis of the Global Enteric Multi-center Study (GEMS) has shown a strong association between diarrhea-causing members of *Enterobacteriaceae* family, such as *E. coli*, and poor nutritional status of the children (<5 years) from countries in Africa and Asia.^47^ We observed *Enterobacteriaceae* tissue-specific (colon vs ileum) colonization patterns, which we hypothesize may be attributed to the interactions among different commensals in an MND environment.^48^, ^49^, ^50^ Previous work from our lab has shown that *Enterobacteriaceae* and *Bacteroidales spp.* grow cooperatively in a protein and iron-deficient environment.^23^^,^^51^ Additionally, increased expression of defensins antimicrobial peptides in ileal mucosa (refer to Table 5) may have limited the growth of *Enterobacteriaceae* in LM ilea. Such site-specific increase in *Enterobacteriaceae* colonization enhanced the infiltration of immune cells, expression of pro-inflammatory cytokines (TNF-α and IL-6), and reduction in anti-inflammatory (IL-10) cytokine expression in colons of LM pups, perhaps via an altered ‘weaning reaction.^52^ Furthermore, the increased recruitment of Ly6C hi monocytes and M1-like macrophages may prime a chronic pro-inflammatory response at colonic mucosa by making the host permissive to co-infections, hence elevating the risk of chronic disorders in adulthood. *Giardia lamblia* persistently colonizes intestines of protein-malnourished mice, thereby promoting mucosal recruitment of cd11b+ monocyte/macrophage populations and co-infection by enteroaggregative *E*. *coli*.^53^

Enteric pathobionts are rampant in children from impoverished and undernourished nations. The molecular mechanisms for increased susceptibility to pathogens remain poorly understood but could be related to mitochondrial dysfunction. Malnutrition has long been known to negatively regulate mitochondrial functions.^35^^,^^54^^,^^55^ A low-protein diet, where 1% of total calories come from protein sources, reduces mitochondrial complexes I, IV, and V expression in mice, causing hepatic steatosis by inhibiting nicotinamide adenine dinucleotide-dependent sirtunin activity.^56^ Mitochondrial dysfunction in the offspring may arise from inefficient vertical transfer of nutrients or via inheritance of epigenetic changes occurring during pregnancy.^54^^,^^57^, ^58^, ^59^, ^60^ In a recent human study conducted on mother-infant pairs in Germany, maternal vitamin B12 and folic acid levels significantly correlated with corresponding micronutrients in the offspring.^61^ Vitamin B12 and folate are important mediators in synthesis of S-adenosylmethionine, an indispensable methyl donor for DNA methylation reactions during epigenetic modifications.^62^ Both maternal zinc and folic acid levels regulate methylation of long interspersed element-1 (LINE-1; a surrogate for assessing global DNA methylation) in the offspring,^63^^,^^64^ which seems to be positively associated with the birth weight and neurological function.^64^^,^^65^ Malnutrition affects mitochondrial function, which disturbs the balance between ROS and antioxidant productions.^35^^,^^54^^,^^55^^,^^66^ Blood samples from children (<5 years) with clinically severe acute malnutrition have relatively lower antioxidant and higher mitochondrial DNA levels.^67^ We observed reduced mitochondrial NADH dehydrogenase levels and increased ROS synthesizing *Nox1* enzyme expression in LM pup colons, which may have contributed to the colonization of *Enterobacteriaceae*. Moreover, inherent abnormality of mitochondrial functioning in C57Bl/6J mice may have further contributed to the increased the levels of colonic mucosa-associated *Enterobacteriaceae* in the corresponding LM pups. This suggests that mitochondria may play a critical role in shaping and establishing the intestinal microbiome. Previous studies have shown that mitochondrial ROS accumulation enhances cytoplasmic NOX1 activity, and hence, increases oxygen free radical generation in human embryonic kidney 293T cells.^68^ Interestingly, facultative anaerobic bacteria, like members of *Enterobacteriaceae*, can utilize H_2_O_2_ to facilitate intimate attachment to intestinal epithelium and subsequent colonization of mucosa.^34^ *Citrobacter rodentium* anaerobically reduces NOX1 generated H_2_O_2_ via cytochrome c peroxidase, to outcompete resident commensals and attach to colonic epithelium in mice during early infection.^69^ It is possible that utilization of ROS by *Enterobacteriaceae*, along with increased abundance of short-chain fatty acid producing bacteria, such as *Lactobacillaceae* and *Enterococcaceae*, may have played a role in minimizing intestinal damage caused by the oxidative environment in LM pups.^70^, ^71^, ^72^ Furthermore, despite normalization of *Nox1* expression upon feeding of CON diet, the abundance of intestinal *Enterobacteriaceae* remained high. Our findings align with the ‘window of opportunity’ and developmental origins of health and disease hypotheses, indicating that the gut microbiota composition is particularly responsive to early-life environmental factors like diet, with reduced plasticity in the microbiome thereafter.^73^^,^^74^ Such differences in *Enterobacteriaceae* attachment were not evident in ex vivo cultures, perhaps attributed to the normal *Nox1* expression and inter-individual differences in the procedure for growing organoids, which likely overshadowed the phenotype.

The newly developed MMND model is highly relevant and broadly applicable to the undernourished children in low- and middle-income countries; however, there are a few limitations. First, pectin was used in LM diet to minimize reabsorption of vitamin B12, which can modify microbial abundances in the gut.^75^ We compensated for this by supplementing CON diet with cellulose, another type of fiber known to affect the microbiome.^76^ In our studies of the gut microbiome, we only focused here on *Enterobacteriaceae*, which are poor fiber fermenters.^77^ Second, meconium samples were used as a proxy for maternal nutritional status from the CHILD study due to the inaccessibility of blood and fecal samples from pregnant mothers. Third, although relative abundances represent important information regarding the composition of the microbiome,^78^ it is possible that changes in *Enterobacteriaceae* measured in the CHILD samples could be due to inhibited growth of other, less abundant bacteria. Lastly, while no overt pathology was seen in the small intestine (primary site for micronutrients absorption) of LM pups; however, the observed proteomic changes in the ilea of LM pups suggest that the MMND model could be used to further interrogate mechanistic changes in the tissue as a result of malnutrition.

Taken together, we developed an intriguing early life MMND model that showed mitochondrial dysfunction as a major contributor to the intestinal blooming of *Enterobacteriaceae* in the offspring. This novel MMND model facilitates research into MMNDs, shedding light on their potential public health implications, which were previously difficult to investigate in human populations.

## Materials and Methods

### Ethics Statement

All studies in mice were conducted as per the Canadian Council on Animal Care regulations and were approved by the University of British Columbia Animal Care Committee (A22-0057/A22-0168). The CHILD Study protocols were approved by the human clinical research ethics boards (H07-03120) at all universities and institutions directly involved with the CHILD cohort (McMaster University, University of British Columbia, the Hospital for Sick Children, University of Manitoba, and University of Alberta).

### Murine Model and Tissue Processing

Seven-week-old C57Bl/6N and C57Bl/6J littermate male and female mice were purchased from Charles Rivers and the Jackson Laboratory (Jax), respectively. C57Bl/6J mice were acquired from the maximum barrier facility at Jax. Based on previous findings from our lab,^39^ 2 experimental approaches were undertaken to develop the early-life MMND model using C57Bl/6N mice: In model 1, mice were randomly assigned to either a CON or an LM diet deficient in vitamin A, vitamin B12, vitamin B9, zinc, and iron (#D18062501I and #D19041709I, respectively; Research Diets Inc) (Table 7). Because gut resident bacteria can synthesize vitamin B12 and mice are coprophagic, pectin fiber was added to LM diet to prevent reabsorption of vitamin B12, whereas cellulose fiber was added to CON diet as a corresponding control.^79^ Next, the mice were mated in trios (2 females/male), and all the pregnant mice remained on their respective diets for a total of 42 ± 2 days, starting from the day of mating until weaning. In model 2, mice were mated in a trio while on a breeder’s diet (#0007689; PicoLab Mouse Diet 20 5058, LabDiet). At day 14 of pregnancy (counted from the day a viscous vaginal plug was visible to confirm mating), mice were randomized and switched to either CON or LM treatment diet. All pregnant mice remained on their respective diets for a total of 28 ± 2 days (from day 14 of pregnancy until weaning). For C57Bl/6J mice experiments, model 2 was followed. In agreement with previous findings from our lab, C57Bl/6J mice were determined to lack culturable levels of commensal *Enterobacteriaceae* in their intestines,^80^ possibly due to variation in hygienic conditions among different barrier facilities at Jax. Microbial shifts during animal transportation and the diverse facility conditions found at various institutes and universities may also impact gut microbiota composition.^81^^,^^82^ Subsequently, female C57Bl/6J mice were orally administered *Enterobacteriaceae* (5 × 10^8^ CFU/100 uL; one gavage 7 days prior to mating) isolated from female C57Bl/6N fecal pellets via plating onto McConkey agar. Mice were given ad libitum access to the diet throughout the experiment. At weaning, murine pups were weighed (g), tail lengths (mm) measured, and later sacrificed (∼ 3 weeks old) to ascertain colon length (mm) and harvest tissues for downstream analysis. For the diet-reversal experiment, model 2 was employed except that, after weaning, both LM and CON pups were put on CON diet (hereafter, LMCON and dCON pups, respectively) for 4 weeks before being euthanized for tissue collection.

**Table 7:** Murine Diet Compositions

Product #	D18062501		D19041709	
	CON diet		LMCON w/ pectin	
	gm%	kcal%	gm%	kcal%
Protein	18.9	20	19.2	20
Carbohydrate	63.1	65	61.7	62
Fat	6.5	15	6.6	15
Total		100		100
kcal/gm	3.77		3.77	

Ingredient	gm	kcal	gm	kcal
Casein	0	0	0	0
Egg white	203	812	203	812
L-cystine	0	0	0	0
Corn starch	346	1384	332.7	1330.8
Maltodextrin 10	45	180	45	180
Dextrose	250	1000	250	1000
Sucrose	0	0	0	0
Cellulose, BW200	75	0	22	0
Inulin	25	25	25	25
Pectin, tic gums	0	0	53	53
Soybean oil	70	630	70	630
Mineral mix S10026	10	0	0	0
Mineral mix S19427 (No Ca, P, K, Zn, or Fe)	0	0	10	0
Dicalcium phosphate	13	0	13	0
Calcium carbonate	5.5	0	5.5	0
Potassium citrate, 1 H2O	16.5	0	16.5	0
Ferric citrate (17.4% Fe)	0	0	0.029	0
Zinc carbonate (52.1% Zn)	0	0	0.004	0
Vitamin mix V10001	10	40	0	0
Vitamin mix V15927 (No vitamin A, folate, or B12)	0	0	10	40
Vitamin mix V15928 (350 IU A, 3 ug B12, 0.11 mg folate)	0	0	0	0
Biotin, 1%	0.4	0	0.4	0
Choline bitartrate	2	0	2	0
Pure red dye #40	0	0	0	0
Pure blue dye #1	0	0	0.05	0
Pure yellow dye #5	0.05	0	0	0
Total	1071.45	4071	1058.183	4071

CON, Control; LM, low-micronutrient; LMCON, LM pups transferred to CON diet.

### 16S rRNA Sequencing of Murine Feces

DNA was extracted using the QIAamp PowerFecal Pro DNA kit (51804, Qiagen) as recommended. The V4 region of bacterial 16s rDNA was amplified using indexed, barcoded primers (515F: GTGCCAGCMGCCGCGGTAA; 806R: GGACTACHVHHHTWTCTAAT). Samples were then pooled, and the library was sequenced on an Illumina MiSeq platform using a NextSeq2000 600 cycle P1 kit. Raw reads were quality-filtered and processed using DADA2 implemented in QIIME2.^83^^,^^84^ Taxonomy was assigned using a Naive Bayes classifier trained on the SILVA 138 database of the 515/806 region at 100% amplicon sequence variants (ASV) cutoffs.^85^ Further filtration was performed in R using the phyloseq package to remove singletons and rarefy samples to a uniform sequencing depth of 8000 reads.^86^ Differential abundance analysis was conducted on unrarefied samples using DESeq2 (version 1.34.0).^87^ Results were visualized using the ggplot2^88^ package in R.

### Determination of Enterobacteriaceae in Intestinal Mucosa

Both colon and ileum were aseptically removed, followed by perfusion with sterile phosphate buffered saline (PBS) without Ca^2+^ and Mg^2+^ (1x; Thermo Fisher Scientific; hereafter PBS) and later, distal tissues (∼1–2 cm) were homogenized in PBS (50 mg/mL). Colonies were grown and quantitated on MacConkey agar (#B212123; BD Biosciences). Data were log-transformed and represented as log_10_ colony forming units (CFU/mL).

### RNA Isolation and Gene Transcription of Pro-inflammatory Mediators

RNA was extracted using RNeasy kit (74104, Qiagen) as recommended. Quantitative polymerase chain reaction (qPCR) was performed using SYBR Green (#208054; Qiagen) as recommended by manufacturer. The primers used for qPCR are listed in Table 8.^89^, ^90^, ^91^, ^92^ *Hypoxanthine-guanine phosphoribosyl transferase* (*Hgprt*) was used as an internal control for normalization.

**Table 8:** List of Primers Used in the Study

Gene	Forward	Reverse
*Tnf-a*	GCCTCTTCTCATTCCTGCTTG	CTGATGAGAGGGAGGCCATT
*Il-6*	ACGGCCTTCCCTACTTCACA	CATTTCCACGATTTCCCAGA
*KC*	AAAAGGTGTCCCCAAGTA	AAGCAGAACTGAACTACCATCG
*Nox 1*	CCTCCTGACTGTGCCAAAGG	ATTTGAACAACAGCACTCACCAA
*Hgprt*	TCAGTCAACGGGGGACATAAA	GGGGCTGTACTGCTTAACCAG

### Cytometric Bead Array Assay

Cytokines were measured in serum samples by flow cytometry using a BD Cytometric Bead Array (CBA) Mouse Inflammation Kit (552364; BD Biosciences) according to the manufacturer’s instructions.

### Histology

Distal ileum and colon tissues were fixed in 10% neutral-buffered formalin and embedded in paraffin. Slides were prepared with 5-micron thick sections and stained commercially at Wax-it Histology Services Inc. Hematoxylin-eosin (H&E) images were taken using BX53 light microscope (Olympus) at 60× magnification in tile-format.

### Intestinal Immune Cell Isolation and Flow Cytometry

Intestinal immune cells were isolated as described previously.^93^ Briefly, intestinal epithelial cells were dissociated from tissues (37 ˚C for 45 minutes) in PBS containing 5 mM Ethylenediaminetetraacetic acid (EDTA; #324506; EMD Millipore) and passed through 70 μM filter for use in proteomics study. Then, the intact pieces of tissue were digested using 5% fetal bovine serum (FBS) (#A3160502; Gibco) in PBS with calcium and magnesium and 0.5 mg/mL collagenase from *Clostridium histolyticum* (#C2139; Sigma-Aldrich). Single cells were collected through 70 μM filters via centrifugation (800 × *g*, 4 °C, 10 minutes), and red blood cells were lysed using ammonium–chloride–potassium lysing buffer (#A1049201; Gibco). For flow cytometry, isolated immune cells were stained with respective antibodies (Table 9) diluted 1:500 in column buffer (ie, 2 mM EDTA, 10 mM HEPES [#15630080; Thermo Fisher Scientific]), 5% (v/v) FBS in PBS (20 minutes at 4 ˚C). After staining, cells were fixed overnight in a 1:1 fix solution of column buffer: 4% PFA. Flow cytometry data were collected with a CytoFLEX (Beckman Coulter) machine and analyzed with FlowJo (Version 10) software.

**Table 9:** List of Antibodies Used in this Study

Cell marker	Fluorophore	Source	Clone	Catalog #
CD45	BV605	BioLegend	30-F11	103139
CD3	e450	BioLegend	17A2	100214
CD4	e506	ThermoFischer	RM4-5	69-0042-82
CD8	PE610	ThermoFischer	53-6.7	61-0081-82
NK1.1	APCe780	ThermoFischer	PK136	47-5941-82
CD19	SB780	ThermoFischer	1D3	78-0193-82
CD11b	e506	ThermoFischer	M1/70	69-0112-82
F4/80	e450	ThermoFischer	BM8	48-4801-82
MHCII	PerCp	BioLegend	M5/114.15.2	107624
Ly6c	PE	ThermoFischer	AL-21	12-5932-82

### Proteomics

Ten micrograms of protein lysate were reduced and alkylated^94^ before running on 10% SDS-PAGE. Proteins in the entire lane were digested overnight in gel with a total of 0.3 μg trypsin-ultra-MS grade (#P8101S; New England BioLabs Inc).^95^ Resulting peptides were cleaned using C-18 STop and Go Extraction (STAGE) tips.^96^ Peptide concentrations were measured using NanoDrop One (Thermo Fisher Scientific), and then approximately 100 ng of peptides from each sample were loaded on Orbitrap Exploris 480 (Thermo Fisher Scientific) coupled to easy nLC 1200 (Thermo Fisher Scientific) with ionopticks’ Aurora series 25 cm × 75 μm C18 1.6-μm column heated to 40 °C. Forty-five minutes of separation was set from 3% to 25% Buffer B (80% acetonitrile, 0.1% formic acid), followed by an additional 15 minutes to reach 35% concentration of buffer B before the necessary column wash. Buffer A consisted of 2% acetonitrile and 0.1% formic acid, and LC flow rate was set at 250 nL/min. Instrument settings were as follows: Instrument polarity was set to positive mode, the spray voltage was set to 1900 V, the ion transfer tube temperature was set to 290 °C, the expected peak width to 15 seconds, the RF lens was set to 50%, and FAIMS was not enabled with data acquisition. Data-dependent mode was set for 20 scans, and the orbitrap resolution was set to 120,000 for full MS and 15,000 for fragment MS, with AGC target set to 100% with 20 ms injection time (IT) for full MS and 50% with auto IT for fragment MS. Profile data type was acquired at full MS level, and centroid at fragment MS level. Intensity threshold for fragmentation was set at 8,000, isolation window for fragmentation at 2 m/z, with normalized collision energy at 28%. Dynamic exclusion was enabled to exclude after 1 time for 45 seconds. Sample batch was randomized before injection.

Acquired data were searched on MaxQuant version 2.1.0.0^97^ against Uniprot’s murine sequences (UP000000589, downloaded April 12, 2019) and common contaminant sequences. Label-free quantitation, iBAQ, and match-between-run options were enabled using peptide mass tolerance of 4.5 ppm, fixed modification of carbamidomethylation of cysteines, and variable modifications of oxidation of methionines and acetylation of protein N-termini. Specific proteolytic cleavages after arginine and lysine with up to 2 missed cleavages were set. The data were filtered for 1% false discovery at protein, peptide, and PSM levels using revert decoy search mode.

### TC7 Cell Culture Experiment

The human colonic epithelial cell line, TC7 (SCC209; Sigma-Aldrich) was cultured in Dulbecco’s Modified Eagle’s Media (DMEM; Thermo Fisher Scientific) and supplemented with 10% FBS, 1% GlutMax (35050-061; Thermo Fisher Scientific) and 1% non-essential amino acids (11140050; Thermo Fisher Scientific) in a humidified environment at 37 °C with 5% CO2. For experiments, cells were treated with Rotenone (R-8875; Sigma-Aldrich) at variable doses (10–100 μM) for 3 hours at 37 °C, followed by infection with *E. coli* Nissle 1917 (isolated from probiotic Mutaflor and later, validated by Sanger sequencing; hereafter *E. coli*). *E. coli* cultures (5 mL) were grown aerobically overnight (∼16 hours) in a shaker incubator (225 rpm at 37 °C) and then, sub-cultured (1:100) before infecting cells at multiplicity of infection (MoI) = 1, for 2 hours. Then, cells were washed, resuspended in 0.1% Triton X-100 (T-9284; Sigma-Aldrich) and plated on Luria-Bertani (LB)-agar medium (#244520; BD Biosciences). Bacterial colonies were counted, log-transformed, and represented as log_10_ CFU/mL.

### CHILD Birth Cohort

The CHILD cohort is a prospective, longitudinal study that enrolled pregnant mothers from 4 cities (Vancouver, Edmonton, Winnipeg, and Toronto) across Canada in their second trimester from 2008 to 2011.^98^ Of 3405 recruited to the General cohort, 77 were ineligible at birth, and 32 withdrew before any maternal or child data were collected, leaving 3296 eligible children with some maternal data. Thirty-two additional subjects withdrew before childbirth, leaving 3264 eligible children commencing the study.

**Inclusion criteria:**

(1) Pregnant women aged 18 years and older (19 in Vancouver);
(2) Residence in reasonable proximity to the delivery hospital;
(3) Able to read, write, and speak English;
(4) Willing to provide informed consent;
(5) Willing to consent to cord blood collection for the study;
(6) Planning to give birth at a designated recruitment center participating hospital;
(7) Infants born at or after 35 weeks;
(8) Able to provide name, address, and telephone numbers of two alternate contact individuals.

**Exclusion criteria:**

(1) Children born with major congenital abnormalities or respiratory distress syndrome (RDS);
(2) Expectation of moving away from a recruitment area within 1 year;
(3) Children of multiple births;
(4) Children resulting from in vitro fertilization;
(5) Children who will not spend at least 80% of nights in the index home;
(6) Children born before 35 weeks gestation

In the CHILD cohort protocol, the first stool passed was collected within 24 hours of birth as the meconium sample. Meconium samples were obtained from a larger cohort that already contained 16s sequencing data as part of a study focused on the impact of prenatal exposures on early infant microbiome maturation and the development of atopic sensitization.^99^ Since meconium begins accumulating by gestational week 16, it offers an excellent snapshot of exposures and nutrients received by the infant from the mother.^100^ Furthermore, comprised of ingested materials, including skin and gut cells, amniotic fluid, vernix and lanugo hair, as well as excreted fetal metabolites, meconium also provides the initial nutrient niche for pioneering bacteria that will establish the nascent microbiota.^101^ Thus, we used meconium to assess intestinal vitamin levels in the children. In this study, we analyzed the meconium metabolome and 3-month infant stool microbiota in a subset of 100 infants.^99^

### Human Stool 16s Sequencing and Preprocessing

The gut microbiota of infants in the CHILD cohort was derived as previously described.^99^^,^^102^ Briefly, the V4 hypervariable region of the 16s rRNA gene was sequenced using universal primers (V4-515f: V4-806r). Paired-end sequences were pre-processed using Dada2 in Qiime2 v.2018.6 (www.qiime2.org).^83^ Taxonomic identity was assigned to the resulting ASVs by alignment to the Greengenes reference (v13.8) database at 99% sequence similarity. Sequences were further filtered in R (version 4.2.1) to remove samples with less than 8000 bp reads and ASVs with less than 10% prevalence before being normalized by relative abundance for downstream analyses.

### Human Meconium Metabolomics

Meconium metabolic profiles were characterized in the CHILD cohort as previously described.^99^ Briefly, 100 mg of meconium from 100 infants were stored at −80 °C and sent to Metabolon, Inc for non-targeted metabolic profiling via their mView Global Metabolomics Profiling Platform using Ultrahigh Performance Liquid Chromatography-Tandem Mass Spectroscopy. A total of 714 different metabolites, including those specific to vitamin metabolism, were identified by automated comparison of the meconium samples to an in-house library of chemical standards that included retention time, molecular weight (m/z), and associated MS spectra and were visually inspected and curated for quality control using software developed at Metabolon. Raw units for each vitamin/co-factor metabolite are defined by area under the curve (AUC).

### Correlating Human Meconium Vitamin Abundance and Enterobacteriaceae Colonization

Of the 714 meconium metabolites quantified by Metabolon, we identified 32 metabolites as belonging to ‘Cofactors and Vitamins’ super pathway (Table 3). Individual and cumulative (‘total vitamins’) raw abundances of metabolites were correlated to specific and aggregated ASVs (by Phyloseq version 1.40.0^86^) of *Enterobacteriaceae*. For the ‘total vitamins’ approach, raw abundances of individual metabolites were first scaled to ensure that higher and lower abundant metabolites had similar influences on the total sum as described previously.^99^ The cumulative AUC sum of all 32 vitamin metabolites were then scaled again to ensure a more succinct range while still reflecting the spread within the 100 samples. Spearman correlations and scatter plot were calculated and visualized via ggpubr (version 0.5.0) and ggplot2 (version 3.4.0).

### Organoid Culture

Murine colonic organoid cultures were grown as previously described.^103^ Briefly, murine colons were chopped into pieces (∼1 cm), and crypts were isolated. The crypts were cultured as hanging drop in Matrigel matrix (356231; Corning) in IntestiCult Organoid Growth Medium (06005; STEMCELL Technologies) for 10 days until the colonoid morphology was apparent. During the experiment, organoids were mechanically disrupted in TrypLE Express Enzyme solution (12604013; Thermo Fisher; 37 °C for 5 minutes) and then plated and cultured in Matrigel-coated (1:49 in PBS) 96-well plate for 7 days. qPCR and *E. coli* attachment assays were performed as described above. In all cases, the organoid media was replaced every 2 to 3 days.

### Statistical Analyses

Analytical data represented as histograms were recorded as mean ± standard deviation and compared using non-parametric 2-sided Mann-Whitney test or Kruskal-Wallis 1-way analysis of variance for multiple group comparisons to control group. Prior to statistical analyses of proteomics data, protein intensity values were log-transformed with zeros converted to missing values. Differential expression analysis was conducted using the limma package in R to test for significant abundance differences between LM and CON pups. To assess the functional roles of the significant proteins, an over-representation analysis was conducted (hypergeometric test) using annotations from GO (Gene Ontology Consortium 2004) and Reactome.^104^ Mouse GO annotations were taken from the org.Mm.eg.db R package (version 3.13.0). GO over-representation was calculated using the GOStats R package. Reactome annotations were taken from the ReactomePA R package (version 1.36.0).^105^ All multiple comparisons were adjusted using the Benjamini-Hochberg correction (q-value), and a P- or q-value of < .05 was considered significant. All statistical analyses were performed with Graph Pad Prism software (Graph Pad version 9.1.0), except for proteomics and CHILD data, where R (version 4.2.1) was used.

## References

[1] Stevens G.A., Beal T., Mbuya M.N.N., Global Micronutrient Deficiencies Research Group (2022). Micronutrient deficiencies among preschool-aged children and women of reproductive age worldwide: a pooled analysis of individual-level data from population-representative surveys. Lancet Glob Health.

[2] Bird J.K., Murphy R.A., Ciappio E.D., McBurney M.I. (2017). Risk of deficiency in multiple concurrent micronutrients in children and adults in the United States. Nutrients.

[3] Semba R.D. (2012). The historical evolution of thought regarding multiple micronutrient nutrition. J Nutr.

[4] Wallace T.C., McBurney M., Fulgoni V.L. (2014). Multivitamin/mineral supplement contribution to micronutrient intakes in the United States, 2007-2010. J Am Coll Nutr.

[5] Committee on World Food Security. High Level Panel of Experts. HLPE#12 R. A report by high level panel of experts on food security and nutrition. 2017. https://www.fao.org/fileadmin/user_upload/hlpe/hlpe_documents/HLPE_Reports/HLPE-Report-12_EN.pdf. Accessed December 15, 2023.

[6] Bailey R.L., West K.P., Black R.E. (2015). The epidemiology of global micronutrient deficiencies. Ann Nutr Metab.

[7] Kaganov B., Caroli M., Mazur A. (2015). Suboptimal micronutrient intake among children in Europe. Nutrients.

[8] Mensink G.B., Fletcher R., Gurinovic M. (2013). Mapping low intake of micronutrients across Europe. Br J Nutr.

[9] Keats E.C., Haider B.A., Tam E., Bhutta Z.A. (2019). Multiple-micronutrient supplementation for women during pregnancy. Cochrane Database Syst Rev.

[10] Cetin I., Buhling K., Demir C. (2019). Impact of micronutrient status during pregnancy on early nutrition programming. Ann Nutr Metab.

[11] Hansu K., Cikim I.G. (2022). Vitamin and mineral levels during pregnancy. Rev Assoc Med Bras (1992).

[12] Maykondo B.K., Horwood C., Haskins L. (2022). A qualitative study to explore dietary knowledge, beliefs, and practices among pregnant women in a rural health zone in the Democratic Republic of Congo. J Health Popul Nutr.

[13] Stinson L.F. (2020). Establishment of the early-life microbiome: a DOHaD perspective. J Dev Orig Health Dis.

[14] Chandra H., Sharma K.K., Tuovinen O.H. (2021). Pathobionts: mechanisms of survival, expansion, and interaction with host with a focus on Clostridioides difficile. Gut Microbes.

[15] Jones H.J., Bourke C.D., Swann J.R., Robertson R.C. (2023). Malnourished microbes: host-microbiome interactions in child undernutrition. Annu Rev Nutr.

[16] Amadi B., Zyambo K., Chandwe K. (2021). Adaptation of the small intestine to microbial enteropathogens in Zambian children with stunting. Nat Microbiol.

[17] Gough E.K., Moulton L.H., Mutasa K. (2020). Sanitation Hygiene Infant Nutrition Efficacy (SHINE) Trial Team. Effects of improved water, sanitation, and hygiene and improved complementary feeding on environmental enteric dysfunction in children in rural Zimbabwe: a cluster-randomized controlled trial. PLoS Negl Trop Dis.

[18] MAL-ED Network Investigators (2017). Relationship between growth and illness, enteropathogens and dietary intakes in the first 2 years of life: findings from the MAL-ED birth cohort study. BMJ Glob Health.

[19] Chae S.A., Son J.S., de Avila J.M. (2022). Maternal exercise improves epithelial development of fetal intestine by enhancing apelin signaling and oxidative metabolism. Am J Physiol Regul Integr Comp Physiol.

[20] Xiao Z., Liu S., Li Z. (2022). The maternal microbiome programs the m(6)A epitranscriptome of the mouse fetal brain and intestine. Front Cell Dev Biol.

[21] Gregorieff A., Stange D.E., Kujala P. (2009). The ets-domain transcription factor Spdef promotes maturation of goblet and paneth cells in the intestinal epithelium. Gastroenterology.

[22] Gardner D.S., Ozanne S.E., Sinclair K.D. (2009). Effect of the early-life nutritional environment on fecundity and fertility of mammals. Philos Trans R Soc Lond B Biol Sci.

[23] Brown E.M., Wlodarska M., Willing B.P. (2015). Diet and specific microbial exposure trigger features of environmental enteropathy in a novel murine model. Nat Commun.

[24] Cichon B., Fabiansen C., Yaméogo C.W. (2016). Children with moderate acute malnutrition have inflammation not explained by maternal reports of illness and clinical symptoms: a cross-sectional study in Burkina Faso. BMC Nutrition.

[25] Patterson G.T., Manthi D., Osuna F. (2021). Environmental, metabolic, and inflammatory factors converge in the pathogenesis of moderate acute malnutrition in children: an observational cohort study. Am J Trop Med Hyg.

[26] Corware K., Yardley V., Mack C. (2014). Protein energy malnutrition increases arginase activity in monocytes and macrophages. Nutr Metab (Lond).

[27] Michael H., Langel S.N., Miyazaki A. (2020). Malnutrition decreases antibody secreting cell numbers induced by an oral attenuated human rotavirus vaccine in a human infant fecal microbiota transplanted gnotobiotic pig model. Front Immunol.

[28] Rodriguez L., Gonzalez C., Flores L. (2005). Assessment by flow cytometry of cytokine production in malnourished children. Clin Diagn Lab Immunol.

[29] Song P., Zhang J., Zhang Y. (2018). Hepatic recruitment of CD11b+Ly6C+ inflammatory monocytes promotes hepatic ischemia/reperfusion injury. Int J Mol Med.

[30] Pang J., Maienschein-Cline M., Koh T.J. (2021). Enhanced proliferation of Ly6C(+) monocytes/macrophages contributes to chronic inflammation in skin wounds of diabetic mice. J Immunol.

[31] Arts R.J., Blok B.A., van Crevel R. (2015). Vitamin A induces inhibitory histone methylation modifications and down-regulates trained immunity in human monocytes. J Leukoc Biol.

[32] Castleman M.J., Dillon S.M., Purba C. (2020). Enteric bacteria induce IFNgamma and granzyme b from human colonic group 1 innate lymphoid cells. Gut Microbes.

[33] Seo B.B., Marella M., Yagi T., Matsuno-Yagi A. (2006). The single subunit NADH dehydrogenase reduces generation of reactive oxygen species from complex I. FEBS Lett.

[34] Crowley S.M., Vallance B.A. (2020). Microbial respiration in the colon: using H(2)O(2) to catch your breath. Cell Host Microbe.

[35] Rodriguez-Cano A.M., Calzada-Mendoza C.C., Estrada-Gutierrez G. (2020). Nutrients, mitochondrial function, and perinatal health. Nutrients.

[36] Dikalov S. (2011). Cross talk between mitochondria and NADPH oxidases. Free Radic Biol Med.

[37] Nabwera H.M., Espinoza J.L., Worwui A. (2021). Interactions between fecal gut microbiome, enteric pathogens, and energy regulating hormones among acutely malnourished rural Gambian children. EBioMedicine.

[38] Vozenilek A.E., Vetkoetter M., Green J.M. (2018). Absence of nicotinamide nucleotide transhydrogenase in C57BL/6J mice exacerbates experimental atherosclerosis. J Vasc Res.

[39] Littlejohn P.T., Bar-Yoseph H., Edwards K. (2023). Multiple micronutrient deficiencies alter energy metabolism in host and gut microbiome in an early-life murine model. Front Nutr.

[40] Sandstrom B. (2001). Micronutrient interactions: effects on absorption and bioavailability. Br J Nutr.

[41] Gu T., Jia X., Shi H. (2022). An evaluation of exposure to 18 toxic and/or essential trace elements exposure in maternal and cord plasma during pregnancy at advanced maternal age. Int J Environ Res Public Health.

[42] Jiang T., Christian P., Khatry S.K. (2005). Micronutrient deficiencies in early pregnancy are common, concurrent, and vary by season among rural Nepali pregnant women. J Nutr.

[43] Pathak P., Kapil U., Kapoor S.K. (2004). Prevalence of multiple micronutrient deficiencies amongst pregnant women in a rural area of Haryana. Indian J Pediatr.

[44] DiTroia S.P., Percharde M., Guerquin M.J. (2019). Maternal vitamin C regulates reprogramming of DNA methylation and germline development. Nature.

[45] Mottram L., Speak A.O., Selek R.M. (2016). Infection susceptibility in gastric intrinsic factor (vitamin B12)-defective mice is subject to maternal influences. mBio.

[46] Hidalgo-Villeda F., Million M., Defoort C. (2023). Prolonged dysbiosis and altered immunity under nutritional intervention in a physiological mouse model of severe acute malnutrition. iScience.

[47] Liu J., Platts-Mills J.A., Juma J. (2016). Use of quantitative molecular diagnostic methods to identify causes of diarrhoea in children: a reanalysis of the GEMS case-control study. Lancet.

[48] Foster K.R., Schluter J., Coyte K.Z., Rakoff-Nahoum S. (2017). The evolution of the host microbiome as an ecosystem on a leash. Nature.

[49] Coyte K.Z., Rakoff-Nahoum S. (2019). Understanding competition and cooperation within the mammalian gut microbiome. Curr Biol.

[50] Coyte K.Z., Schluter J., Foster K.R. (2015). The ecology of the microbiome: networks, competition, and stability. Science.

[51] Huus K.E., Bauer K.C., Brown E.M. (2020). Commensal bacteria modulate immunoglobulin A binding in response to host nutrition. Cell Host Microbe.

[52] Al Nabhani Z., Dulauroy S., Marques R. (2019). A weaning reaction to microbiota is required for resistance to immunopathologies in the adult. Immunity.

[53] Bartelt L.A., Bolick D.T., Mayneris-Perxachs J. (2017). Cross-modulation of pathogen-specific pathways enhances malnutrition during enteric co-infection with Giardia lamblia and enteroaggregative Escherichia coli. PLoS Pathog.

[54] Bomer N., Pavez-Giani M.G., Grote Beverborg N. (2022). Micronutrient deficiencies in heart failure: Mitochondrial dysfunction as a common pathophysiological mechanism?. J Intern Med.

[55] Reusens B., Theys N., Remacle C. (2011). Alteration of mitochondrial function in adult rat offspring of malnourished dams. World J Diabetes.

[56] Hu G., Ling C., Chi L. (2022). The role of the tryptophan-NAD + pathway in a mouse model of severe malnutrition induced liver dysfunction. Nat Commun.

[57] Luo S., Valencia C.A., Zhang J. (2018). Biparental inheritance of mitochondrial DNA in humans. Proc Natl Acad Sci U S A.

[58] Zuccarello D., Sorrentino U., Brasson V. (2022). Epigenetics of pregnancy: looking beyond the DNA code. J Assist Reprod Genet.

[59] Hales C.N., Barker D.J. (2001). The thrifty phenotype hypothesis. Br Med Bull.

[60] Koemel N.A., Skilton M.R. (2022). Epigenetic aging in early life: role of maternal and early childhood nutrition. Curr Nutr Rep.

[61] Reischl-Hajiabadi A.T., Garbade S.F., Feyh P. (2022). Maternal vitamin B(12) deficiency detected by newborn screening-evaluation of causes and characteristics. Nutrients.

[62] Mentch S.J., Locasale J.W. (2016). One-carbon metabolism and epigenetics: understanding the specificity. Ann N Y Acad Sci.

[63] Rerkasem A., Nantakool S., Wilson B.C. (2022). Associations between maternal plasma zinc concentrations in late pregnancy and LINE-1 and Alu methylation loci in the young adult offspring. PLoS One.

[64] Fryer A.A., Nafee T.M., Ismail K.M. (2009). LINE-1 DNA methylation is inversely correlated with cord plasma homocysteine in man: a preliminary study. Epigenetics.

[65] Joubert B.R., den Dekker H.T., Felix J.F. (2016). Maternal plasma folate impacts differential DNA methylation in an epigenome-wide meta-analysis of newborns. Nat Commun.

[66] Harvey A.J. (2019). Mitochondria in early development: linking the microenvironment, metabolism and the epigenome. Reproduction.

[67] Saha D., Mehndiratta M., Aaradhana (2022). Oxidative stress, mitochondrial dysfunction, and premature ageing in severe acute malnutrition in under-five children. Indian J Pediatr.

[68] Lee S.B., Bae I.H., Bae Y.S., Um H.D. (2006). Link between mitochondria and NADPH oxidase 1 isozyme for the sustained production of reactive oxygen species and cell death. J Biol Chem.

[69] Miller B.M., Liou M.J., Zhang L.F. (2020). Anaerobic respiration of NOX1-derived hydrogen peroxide licenses bacterial growth at the colonic surface. Cell Host Microbe.

[70] Caetano M.A.F., Castelucci P. (2022). Role of short chain fatty acids in gut health and possible therapeutic approaches in inflammatory bowel diseases. World J Clin Cases.

[71] Deleu S., Machiels K., Raes J. (2021). Short chain fatty acids and its producing organisms: an overlooked therapy for IBD?. EBioMedicine.

[72] Fusco W., Lorenzo M.B., Cintoni M. (2023). Short-chain fatty-acid-producing bacteria: key components of the human gut microbiota. Nutrients.

[73] Thriene K., Michels K.B. (2023). Human gut microbiota plasticity throughout the life course. Int J Environ Res Public Health.

[74] Littlejohn P., Finlay B.B. (2021). When a pandemic and an epidemic collide: COVID-19, gut microbiota, and the double burden of malnutrition. BMC Med.

[75] Blanco-Perez F., Steigerwald H., Schulke S. (2021). The dietary fiber pectin: health benefits and potential for the treatment of allergies by modulation of gut microbiota. Curr Allergy Asthma Rep.

[76] Kim Y., Hwang S.W., Kim S. (2020). Dietary cellulose prevents gut inflammation by modulating lipid metabolism and gut microbiota. Gut Microbes.

[77] Williams B.A., Grant L.J., Gidley M.J., Mikkelsen D. (2017). Gut fermentation of dietary fibres: physico-chemistry of plant cell walls and implications for health. Int J Mol Sci.

[78] Zeng M.Y., Inohara N., Nunez G. (2017). Mechanisms of inflammation-driven bacterial dysbiosis in the gut. Mucosal Immunol.

[79] Ghosh S., Sinha J.K., Putcha U.K., Raghunath M. (2016). Severe but not moderate vitamin B12 deficiency impairs lipid profile, induces adiposity, and leads to adverse gestational outcome in female C57BL/6 mice. Front Nutr.

[80] Serapio-Palacios A., Woodward S.E., Vogt S.L. (2022). Type VI secretion systems of pathogenic and commensal bacteria mediate niche occupancy in the gut. Cell Rep.

[81] Montonye D.R., Ericsson A.C., Busi S.B. (2018). Acclimation and Institutionalization of the mouse microbiota following transportation. Front Microbiol.

[82] Cazares-Olivera M., Miroszewska D., Hu L. (2022). Animal unit hygienic conditions influence mouse intestinal microbiota and contribute to T-cell-mediated colitis. Exp Biol Med (Maywood).

[83] Bolyen E., Rideout J.R., Dillon M.R. (2019). Reproducible, interactive, scalable and extensible microbiome data science using QIIME 2. Nat Biotechnol.

[84] Callahan B.J., McMurdie P.J., Rosen M.J. (2016). DADA2: high-resolution sample inference from Illumina amplicon data. Nat Methods.

[85] Pruesse E., Quast C., Knittel K. (2007). SILVA: a comprehensive online resource for quality checked and aligned ribosomal RNA sequence data compatible with ARB. Nucleic Acids Res.

[86] McMurdie P.J., Holmes S. (2013). phyloseq: an R package for reproducible interactive analysis and graphics of microbiome census data. PLoS One.

[87] Love M.I., Huber W., Anders S. (2014). Moderated estimation of fold change and dispersion for RNA-seq data with DESeq2. Genome Biol.

[88] Wickham H. (2016).

[89] Yamakawa I., Kojima H., Terashima T. (2011). Inactivation of TNF-alpha ameliorates diabetic neuropathy in mice. Am J Physiol Endocrinol Metab.

[90] De Filippo K., Henderson R.B., Laschinger M., Hogg N. (2008). Neutrophil chemokines KC and macrophage-inflammatory protein-2 are newly synthesized by tissue macrophages using distinct TLR signaling pathways. J Immunol.

[91] Eissa N., Kermarrec L., Hussein H. (2017). Appropriateness of reference genes for normalizing messenger RNA in mouse 2,4-dinitrobenzene sulfonic acid (DNBS)-induced colitis using quantitative real time PCR. Sci Rep.

[92] Moll F., Walter M., Rezende F. (2018). NoxO1 controls proliferation of colon epithelial cells. Front Immunol.

[93] Boutin R.C., Petersen C., Woodward S.E. (2021). Bacterial-fungal interactions in the neonatal gut influence asthma outcomes later in life. Elife.

[94] Goodman J.K., Zampronio C.G., Jones A.M.E., Hernandez-Fernaud J.R. (2018). Updates of the in-gel digestion method for protein analysis by mass spectrometry. Proteomics.

[95] Shevchenko A., Wilm M., Vorm O., Mann M. (1996). Mass spectrometric sequencing of proteins silver-stained polyacrylamide gels. Anal Chem.

[96] Ishihama Y., Rappsilber J., Andersen J.S., Mann M. (2002). Microcolumns with self-assembled particle frits for proteomics. J Chromatogr A.

[97] Tyanova S., Temu T., Cox J. (2016). The MaxQuant computational platform for mass spectrometry-based shotgun proteomics. Nat Protoc.

[98] Moraes T.J., Lefebvre D.L., Chooniedass R., CHILD Study Investigators (2015). The Canadian healthy infant longitudinal development birth cohort study: biological samples and biobanking. Paediatr Perinat Epidemiol.

[99] Petersen C., Dai D.L.Y., Boutin R.C.T. (2021). A rich meconium metabolome in human infants is associated with early-life gut microbiota composition and reduced allergic sensitization. Cell Rep Med.

[100] Skelly C.L., Zulfiqar H., Sankararaman S. (2023).

[101] Houghteling P.D., Walker W.A. (2015). Why is initial bacterial colonization of the intestine important to infants' and children's health?. J Pediatr Gastroenterol Nutr.

[102] Fehr K., Moossavi S., Sbihi H. (2020). Breastmilk feeding practices are associated with the co-occurrence of bacteria in mothers' milk and the infant gut: the CHILD Cohort Study. Cell Host Microbe.

[103] Holani R., Babbar A., Blyth G.A.D. (2020). Cathelicidin-mediated lipopolysaccharide signaling via intracellular TLR4 in colonic epithelial cells evokes CXCL8 production. Gut Microbes.

[104] Gillespie M., Jassal B., Stephan R. (2022). The reactome pathway knowledgebase 2022. Nucleic Acids Res.

[105] Yu G., He Q.Y. (2016). ReactomePA: an R/Bioconductor package for reactome pathway analysis and visualization. Mol Biosyst.

